# The OptiBreech Trial Feasibility Study: A Qualitative Inventory of the Roles and Responsibilities of Breech Specialist Midwives

**DOI:** 10.1111/jmwh.13728

**Published:** 2025-02-01

**Authors:** Siân M. Davies, Alice Hodder, Shawn Walker, Natasha Bale, Honor Vincent, Tisha Dasgupta, Alexandra Birch, Keelie Piper, Sergio A. Silverio

**Affiliations:** ^1^ Department of Women & Children's Health King's College London London United Kingdom; ^2^ Chelsea and Westminster Hospital NHS Foundation Trust London United Kingdom; ^3^ University College London Hospitals NHS Foundation Trust London United Kingdom; ^4^ NHS Lothian Edinburgh United Kingdom; ^5^ Department of Midwifery and Allied Health Staffordshire University Staffordshire United Kingdom; ^6^ School of Healthcare Leicester University Leicester United Kingdom

**Keywords:** breech specialist midwife, collaborative care, intrapartum care, OptiBreech, template analysis, vaginal breech birth

## Abstract

**Background:**

The safety of vaginal breech birth is associated with the skill and experience of professionals in attendance, but minimal training opportunities exist. OptiBreech collaborative care is an evidence‐based care bundle, based on previous research. This care pathway is designed to improve access to care and the safety of vaginal breech births, when they occur, through dedicated breech clinics and intrapartum support. This improved process also enhances professional training. Care coordination is accomplished in most cases by a key breech specialist midwife on the team. The goal of this qualitative inventory was to describe the roles and tasks undertaken by specialist midwives in the OptiBreech care implementation feasibility study.

**Methods:**

Semistructured interviews were conducted with OptiBreech team members (17 midwives and 4 obstetricians; N = 21), via video conferencing software. Template analysis was used to code, analyze, and interpret data relating to the roles of the midwives delivering breech services. Tasks identified through initial coding were organized into 5 key themes in a template, following reflective discussion at weekly staff meetings and stakeholder events. This template was then applied to all interviews to structure the analysis.

**Results:**

Breech specialist midwives functioned as change agents. In each setting, they fulfilled similar roles to support their teams, whether this role was formally recognized or not. We report an inventory of tasks performed by breech specialist midwives, organized into 5 themes: care coordination and planning, service development, clinical care delivery, education and training, and research.

**Discussion:**

Breech specialist midwives perform a consistent set of roles and responsibilities to co‐ordinate care throughout the OptiBreech pathway. The inventory has been formally incorporated into the OptiBreech collaborative care logic model. This detailed description can be used by employers and professional organizations who wish to formalize similar roles to meet consistent standards and improve care.

## INTRODUCTION

Breech presentation at term (from 37 weeks’ gestation) occurs in 4% of pregnancies.^1^ It is associated with a higher risk of adverse neonatal outcomes, compared with fetuses remaining in cephalic presentation throughout the third trimester. Poor maternal outcomes also occur more frequently, through increased rates of surgical birth and birth trauma.^2^ Outcomes improve when care is provided by clinicians with skill and experience facilitating vaginal breech birth (VBB).^1^, ^3^

**QUICK POINTS**

✦ In qualitative research with 13 sites implementing the OptiBreech care pathway, the role of midwives functioning as specialists was pivotal in leading and coordinating OptiBreech services.
✦ This article reports a formal inventory of the roles and tasks associated with breech specialist midwives in the OptiBreech feasibility studies, as described by the midwives and their colleagues in qualitative interviews.
✦ The tasks and responsibilities of breech specialist midwives include care coordination and planning, service development, clinical care delivery, education and training, and research.
✦ Defining the breech specialist midwife role helps to standardize implementation and enable rigorous testing to determine its effectiveness.

In the United Kingdom and the United States, less than 5% of term breech fetuses are born via VBB.^4^, ^5^ Breech presentation is most often managed with an attempted external cephalic version (ECV) or cesarean birth (CB).^1^, ^3^, ^6^, ^7^ Low rates of VBB have resulted in a lack of training opportunities.^8^, ^9^, ^10^, ^11^ Yet due to maternal choice and undiagnosed breech presentation in labor, VBB continues to occur, with increased vulnerability to poor outcomes.^12^, ^13^, ^14^ Twelve percent of National Health Service (NHS) obstetric litigation costs for cerebral palsy in the United Kingdom relate to poorly managed VBB, diagnosed late in labor, despite representing only 0.3% of total births.^15^

Although the opportunity for VBB attended by experienced providers is difficult to obtain, demand for this option continues in many high‐income countries.^13^, ^14^, ^16^, ^17^ When the option of a physiologic hospital birth is inaccessible, some women choose to plan higher‐risk out‐of‐hospital births.^18^, ^19^, ^20^, ^21^

### OptiBreech Collaborative Care

Research to improve the safety of VBB began in response to women requesting more equitable access to the option of a safe‐as‐possible VBB.^13^, ^14^ OptiBreech collaborative care was then developed as an optimized care pathway for women pregnant with a breech‐presenting fetus at term. Evidence‐based practices are bundled to improve access to all care options (VBB, ECV, and CB) and generate VBB expertise among staff.^22^, ^23^, ^24^ Feasibility and pilot work to test the effectiveness of the care bundle was funded by the UK National Institute for Health and Care Research (NIHR) in 2020.^8^, ^23^

**Figure 1:**
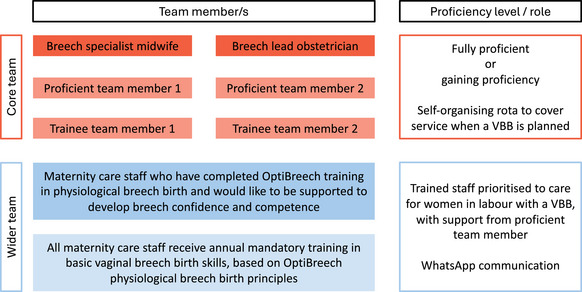
Typical OptiBreech Team Model Abbreviation: VBB, vaginal breech birth.

OptiBreech care is delivered through a dedicated clinic and intrapartum care team, coordinated by a breech specialist midwife (BSM) in collaboration with a breech lead obstetrician‐gynecologist.^25^, ^26^ A small, experienced team provides continuity for women and professionals (Figure 1). Their role is to train and support the wider team to implement the OptiBreech Physiologic Breech Birth Algorithm (Supporting Information: Appendix S1).^27^, ^28^

During implementation and feasibility testing of the OptiBreech care model,^8^ qualitative research among women and clinicians indicated midwives leading and coordinating OptiBreech services were a key component of success.^11^, ^25^ These service leaders were referred to by women and their colleagues as *breech specialists*. The aim of this research was to report a formal inventory of the roles and tasks associated with BSMs in the OptiBreech feasibility studies, as described by the midwives and their colleagues in qualitative interviews.

## METHODS

### Qualitative Approach and Research Paradigm

A template analysis of interview data was the qualitative method chosen to guide our analysis of the BSM role within OptiBreech services.^29^ A template analysis is a pragmatic approach to organizing a data set, beginning with a few a priori codes thought to be important, organizing these into a template, and refining this template through several iterations of analytical application to data, quality checks, and reflexive discussions. Our approach to the overall project was guided by the paradigm of critical realism.^30^ This influenced our analytical approach, in which we sought to describe what was experienced and observed by our participants, while remaining alert to the underlying causal mechanisms and contextual factors that may impact these realities, including our own interpretations of them.

### Researcher Characteristics and Reflexivity

The research team included 3 midwives (S.W., A.B., N.B.), 3 student midwives (A.H., H.V., K.C.), 2 nonclinical research assistants (S.M.D., T.D.), a patient and public involvement and engagement (PPIE) lead (S.M.D.), and a methodological expert (S.A.S.). Coding and analysis for this study was conducted by S.M.D., S.W., H.V., and A.H. S.W. is an experienced BSM who has formally held that role and led the overall OptiBreech project. To maintain researcher objectivity, no members of the research team were interviewed as part of the study. One interview was carried out by an author (S.W.). All other interviews were carried out by OptiBreech members who did not analyze the results or write the article. One clinical and one nonclinical member of the team had personal experience of breech pregnancy. Other members who conducted interviews had minimal personal experience of breech care.

### Context

The study was conducted during an implementation feasibility study (OptiBreech 1) beginning in 2021, undertaken in preparation for a clinical trial.^8^ Clinical teams in 13 NHS hospitals aimed to ensure all planned VBBs were attended by a provider who had completed enhanced training and met proficiency criteria (attendance at a minimum of 10 VBBs). Insights from this implementation study were used to refine the program theory and logic model for the complex intervention now called OptiBreech collaborative care, which was subsequently tested in a pilot randomized trial.^23^

### Sampling Strategy

We used a maximum variation purposeful sampling strategy to explore the acceptability of the intervention for different stakeholders, in different settings.^31^ Consent to be approached and to participate in an interview was integrated into a staff survey administered via Jisc online survey software. The survey also asked about proficiency related to VBB. Principal investigators (PIs) at each site identified further participants based on their involvement with VBBs within the study.

Sample size was guided by informational power, which indicates that when a sample holds more information, relevant for the actual study, fewer participants are needed.^32^ A total of 21 staff were interviewed. Participants came from all 13 participating sites. They included 9 PIs and 12 additional members of staff who had been involved with one or more births on the study. Seventeen participants were midwives, and 4 were obstetrician‐gynecologists. Participants reported career total VBBs attended (range 1‐50, median 6) and VBBs attended within the past year (range 0‐4, median 2).

### Ethical Issues

The OptiBreech 1 study was reviewed and approved by the East of England Cambridgeshire and Hertfordshire Research Ethics Committee (20/EE/0287, IRAS 268668). Previously obtained online consent from participants was confirmed verbally at the start of each interview. Online consent, interview audiovisual, and transcript data were stored on the sponsors’ secure online storage network. Once transcribed, interviews were de‐identified to prevent identification of clinicians or setting.

### Data Collection Methods

Interviews averaged 39 minutes (range, 18‐68 mins) and were conducted between June 17, 2021, and June 16, 2022.

### Data Collection Instruments

Semistructured interview guides were developed to answer the question, “What factors affect the acceptability of proficient breech team care for staff and those receiving care?” Questions were based on component constructs in the theoretical framework for acceptability, including affective attitude, burden, ethicality, intervention coherence, opportunity costs, perceived effectiveness, and self‐efficacy.^33^

In early interviews with OptiBreech staff members, repeated references were made to key midwives working in sites where VBBs were regularly supported. These references were echoed in previously reported, simultaneously occurring interviews with women who planned VBBs within the study.^23^ This was unexpected, as the original protocol suggested, but did not require, that sites establish teams of 5 midwives and 5 obstetrician‐gynecologists, with 1 lead each. Instead, in sites successfully supporting VBBs, most care was given or coordinated by a breech lead midwife.^25^ This midwife worked in collaboration with a breech lead obstetrician‐gynecologist and other team members. However, their leadership ensured the breech services and research were delivered. Because their contribution was so pivotal, the interview guide was revised to elicit more exploration of this role (see Supporting Information Appendix S2).

### Data Collection Technologies and Data Processing

Audiovisual interviews were recorded via Microsoft Teams video conferencing software. Recorded interviews were transcribed using either a General Data Protection Regulation–compliant third‐party transcription service or automatic transcription within the software, with verification by a member of the research team. Pseudonymization and de‐identification of excerpts were performed following transcription and prior to analysis.

### Data Analysis

De‐identified transcripts were coded independently by S.M.D., S.W., A.H., and H.V. using NVivo 12 qualitative data analysis software. Data were analyzed iteratively, between June 2021 and September 2023. Reflective discussions occurred throughout this period.

Template analysis methods structured the analysis, with *roles of breech specialist midwives* used as the a priori theme.^29^ The process included (1) refamiliarization with the data, (2) preliminary coding in a subset of interviews, (3) organization of themes to create an initial coding template, (4) applying and developing the template in further interviews, (5) initial summary and interpretation of the data, (6) presenting these findings to stakeholders in a public meeting to check resonance, and (7) final interpretation.

Based on the first 6 interviews, we initially coded and categorized all tasks that were described by participants as being fulfilled by BSMs. We then developed these categories into a template and applied this to subsequent interviews. Further refinements occurred following reflective discussions to ensure the template was complete and fit well across all interviews. The refined template consisted of 5 thematic categories, each containing related tasks. This was then applied to the complete data set, independently, by S.M.D., H.V., and A.H., and final refinements were made.

Particular attention was paid to comparing the roles of BSMs as perceived by their colleagues with their own perceptions. NVivo coding matrices were used to facilitate this, using profession (midwife or obstetrician) and specialist role (yes or no). Results were discussed among the research team, and the final list of roles was agreed on. Results were shared at an online public meeting, which contributed to our interpretation and discussion.

### Techniques to Enhance Trustworthiness

Interviews were conducted as part of an observational study that also collected data about recruitment rates and outcomes, reported separately.^8^ During analysis, we triangulated our interpretations with observational data by analyzing some variables according to whether a BSM was active within that setting.^8^, ^25^ We also triangulated these findings with those from interviews with women participating in the study.^25^

Reflective discussions involved additional members of the OptiBreech research team. Results were also presented at a British Intrapartum Care Society annual conference and in a public stakeholder meeting. This involved members of clinical OptiBreech teams at participating sites, other NHS staff, and women who had used NHS services during their breech pregnancies, to ensure our interpretations resonated with a wide range of stakeholders.

## RESULTS

**Figure 2:**
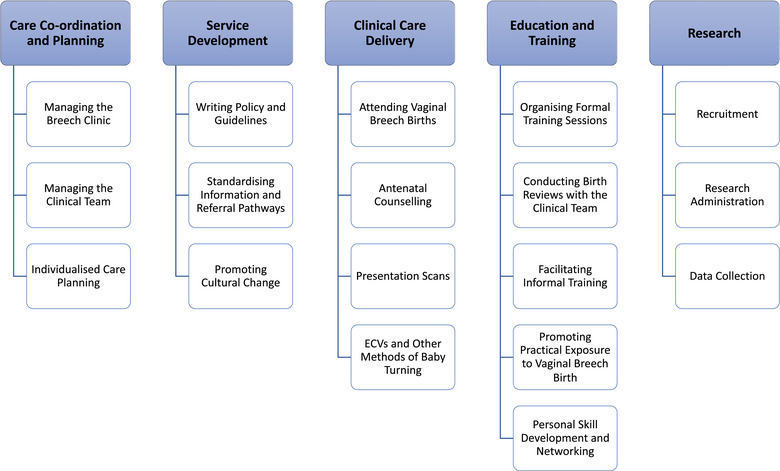
Structure of Breech Specialist Midwife Role Abbreviation: ECV, external cephalic version.

The tasks and responsibilities of BSMs within OptiBreech collaborative care fall into 5 thematic categories: (1) care coordination and planning, (2) service development, (3) clinical care delivery, (4) education and training, and (5) research (Figure 2). Exemplary quotes from each theme can be found in Table 1 with an extended collection included in Supporting Information: Appendix S3.

**Table 1:** Exemplary Quotes from Interview Transcripts

Theme	Quotes
Care coordination and planning	*I think the clinic is gonna be the biggest aspect. I think once we get a clinic up and running, because that's a lot of what I spend my time doing is trying to contact women, arrange when we can meet… So if I've got, you know, I've got a clinic and I could just arrange women to see them there*. (Midwife, specialist)
Service development	*So I've had a couple of meetings with the sonographers, and I've shared with them the policy and the pathway, because I definitely felt that was an area that might have been missing out on the information. And actually, sonographers are often the first people to diagnose a breech birth, and if they straightaway say, Oh, you're gonna have to have a caesarean now, that fear or that seed is already sowed. So I've really tried to work with the sonographers to say, “Just refer the women to the clinic,” so the sonographers directly refer women to us now. So that's helped quite a lot*. (Midwife, specialist)
Clinical care delivery	*I'm currently the only person who meets the proficiency criteria. So I've spent a lot of time on call. And that isn't sustainable in the long run. But I'm kind of viewing it as a process. I'm the thin edge of the wedge, if you will*. (Midwife, specialist)
Education and training	*So went straight back from that, re‐jigged all of our mandatory education for our multi‐professional team and introduced physiological breech birth for the whole multi‐professional team*. (Midwife, specialist) *But in terms of just being with this team, like the WhatsApp, we keep sharing information there. So we're sharing all of the things that we're learning constantly and even people who are not directly involved in care are getting experience about the cases that are going on and I think that is helping, yeah*. (Midwife, specialist)
Research	*It's the first time I've been a PI. So that's been a little bit scary and a little bit—and I have to say I do feel a little bit like a fish out of water. So things like the paperwork side of it, all the logs you've got to keep and making sure that that's all—all those things are done and that apprehension, I suppose sometimes puts me off a little bit, doing some of the stuff I want to—to put the service in and I can put a service in. But then doing all the paperwork for the research side of things, like a different entity, making sure that's all done properly. So that's not been easy to navigate. But I suppose it just comes with experience*. (Midwife, specialist)

Abbreviation: PI, principal investigator.

### Care Coordination and Planning

Some BSMs worked in a dedicated breech clinic. BSMs managed clinic activities, such as overseeing referrals into the service, scheduling appointments, providing antenatal counseling, developing care plans, and coordinating ECVs. They also oversaw the operational management of the clinic, such as arranging the funding, venue, timings, and staffing. BSMs often ran clinics alongside a coassigned obstetrician‐gynecologist, although sometimes the day‐to‐day running was delegated to other breech team members. Having both an obstetric and midwifery presence in the clinic, or midwifery‐led counseling with access to an obstetrician‐gynecologist, contributed to collaborative care planning and greater acceptance of the service among the wider, multidisciplinary team.

In sites where the clinic had not been formalized, it was seen as an essential part of future planning. Dedicated clinics gave BSMs the time and space to have oversight of the service and improved accessibility for service users, as referrals were more consistently made to a centralized clinic.

BSMs developed individualized care plans in partnership with women. For women requesting a planned VBB, the BSMs explained what would happen during labor, including during any potential complications, recommended guidelines, and why these were in place. BSMs mediated communication and understanding between women and the wider multidisciplinary team. They shared and tried to ensure care plans were consistently followed. BSMs received and responded to concerns about women's care choices from colleagues as well as concerns about unwanted intervention or lack of support from women.

Importantly, BSMs coordinated and managed the team of clinicians caring for VBBs. This involved creating formal or informal on‐call lists and collaborating with management to ensure that care was provided by trained OptiBreech team members.

Regular breech team meetings and ad hoc reflective sessions led by BSMs provided a space to share challenges and learnings. These meetings allowed team members to build interprofessional relationships, which improved care delivery. BSMs built confidence among team members by encouraging others to take on responsibility while remaining present for advice and reassurance. Participants saw having a well‐coordinated and supported team as key in sustainably growing the service so that primary responsibility for OptiBreech delivery did not solely fall on the shoulders of the BSM.

### Service Development

BSMs initiated change in the way the organization collectively delivered breech care. They updated existing local guidelines and policies, which influenced education and clinical care. This was seen as an important initial step in implementing the service, as it demonstrated backing from trust leadership.

Staff observed that information provided outside the OptiBreech service was often varied and lacked objectivity, with women receiving conflicting information from different clinicians. Formal clinic referral pathways helped address this concern, although adherence was not always consistent. During service setup, BSMs promoted care referral guidelines, liaising directly with sonography, obstetric, and community midwifery teams.

Participants described working in settings where, although guidelines supported offering the choice of VBB to women, institutional and professional culture often did not. Even when cultures were supportive, OptiBreech care introduced new concepts and techniques for the management of VBBs, which also required cultural change.

Multidisciplinary team education and training were seen as a key way of building support. Resistance to VBB came from a fear of poor outcomes; education sessions provided a space to address these fears and instill confidence. This included sharing videos and stories of successful VBBs.

Building relationships across the team also promoted culture change. BSMs especially sought to build relationships with obstetric consultants and senior midwives whose support impacted how OptiBreech was seen by the rest of the obstetric team. Perception of the change agent as a *specialist* was described as a key enabler. This primarily came from having experience with VBB but also from formally achieving the title of specialist.

The BSM's role as a team leader rather than a single individual also supported cultural change. Team members acted as breech champions who provided expertise and were generally positive about breech, for example, in informal conversations with colleagues, or role modelling using the OptiBreech algorithm in birth rooms. One BSM described breech team members as “resources” for spreading expertise, and another described them as “spokes across the service to be little beacons of advice and positivity around vaginal breech births.”

### Clinical Care Delivery

Specialists delivered clinical care that required advanced practice competencies for midwives. In the early days of building a service, BSMs were often the primary on‐call VBB provider. This was sometimes because they were the only team member with significant experience, or they themselves sought to consolidate their skills to lead the service. In the short term, this was accepted as necessary while other breech team members developed their OptiBreech intrapartum skills, and sustainable during initial setup. All BSMs expected this arrangement to evolve as team experience grew, but our analysis only covered the first year of the service.

BSMs provided antenatal counseling to women with a breech presentation on care options. Staff expressed the view that when counseling was provided by a clinician knowledgeable and experienced in VBB, it helped to ensure this option was offered in a balanced way. They stressed the importance of using evidence‐based information to promote women's autonomous decision‐making and the significance of building trust and confidence in women's choices.

Fetal presentation scans and ECVs were sometimes performed by BSMs and sometimes coordinated through the breech clinic with obstetrician‐gynecologists or ultrasonographers. It was perceived as important to train specialist midwives in these skills to promote continuity of care within the service. BSMs also advised women about other methods of encouraging the fetus to assume a cephalic presentation, such as using moxibustion, acupuncture, or different maternal postural techniques.

### Education and Training

Education and training was a major part of the BSM's role, not only in scaling up the breech team but also in building confidence and support for VBB within the wider multidisciplinary team. BSMs contributed OptiBreech material to mandatory training sessions. They organized study days and oversaw completion of the OptiBreech online training package. In team meetings and drop‐in sessions, they ran through the OptiBreech algorithm and facilitated practice of maneuvers. They encouraged team members to run their own training sessions to consolidate skill acquisition. Specialists reviewed births with the clinical team. These reviews were described as essential in supporting colleagues to integrate learning.

Many participants spoke about the BSM's role in facilitating informal training. This involved fostering a learning culture within the breech team by circulating training material and experience sharing via team WhatsApp groups or in team meetings. Across the wider team, BSMs and breech team members were perceived as experts in VBB, which encouraged staff members to approach them with questions.

Transcripts consistently emphasized the significance of practical exposure to VBB for staff training. BSMs described attending VBBs to support staff to facilitate their learning experience and were consistently available either in person or by telephone. More experienced team members were often scheduled with less experienced members to attend VBBs together. Videos from the OptiBreech online library were available to promote exposure to births for those who could not attend in person.

BSMs continuing personal development was seen as essential. This included attending VBBs to develop intrapartum skillsets, independently reading about breech care, and completing OptiBreech competency training. Participants described networking with other OptiBreech sites so they could seek advice beyond experiences at their own site. This involved attending OptiBreech webinars and using intertrust WhatsApp groups to share experiences and expertise.

### Research

Successful implementation in the OptiBreech feasibility studies was influenced by multiple aspects of the BSMs’ roles. Sites with BSMs attracted clients planning VBBs, which resulted in higher rates of prospective recruitment of women to the OptiBreech study, and consequently more rapid consolidation of learning.

BSMs served as PIs for the OptiBreech study. This involved leading the study within their hospital: recruiting and consenting participants, overseeing implementation of the protocol, and collecting data from births and entering the data into OptiBreech Case Report Forms. This was a new skill for all investigators and contributed to research capacity development within the local workforce.

## DISCUSSION

Within the context of OptiBreech collaborative care, individuals functioning in the role of BSM play a pivotal role in service transformation and the provision of safe, standardized care. Leadership is a key component of culture change found in the management, quality improvement, and implementation science literatures.^34^, ^35^, ^36^ Specifically, midwifery leadership has been shown to be vital in supporting change that promotes physiology. Having BSMs in leadership positions within hospital settings is an important factor toward facilitating culture change.

The BSM role may be defined as a midwife who co‐ordinates perinatal care for women related to breech presentation and contributes to multidisciplinary leadership and development of the breech care pathway. The tasks and responsibilities of BSM encompass aspects of care planning and coordinating, clinical care delivery, education and training, service development, and research. We have incorporated this inventory into the OptiBreech collaborative care package, program theory, and logic model for the purpose of further evaluating the impact of breech specialist roles on clinical outcomes. This inventory can also be used by employers and professional organizations who wish to formalize similar roles. In the United Kingdom, there are increasing calls for regulation of advanced practitioner roles in midwifery.^37^ Regulation of midwife specialist and extended roles in care for clients with breech presentation would require clear and justified standards,^38^ to which this research contributes.

A key difference between standard care and OptiBreech services is that most tasks within OptiBreech care are performed by a BSM or with their support, guidance, and leadership. This resonates with previous research describing breech specialists’ “generative expertise” with a service, that is, how expertise develops and is best mobilized to generate skill and competence with VBB. Consolidating already necessary roles into a coordinated pathway contributes to building staff expertise across the service.

The signal effect for OptiBreech collaborative care on clinical safety outcomes is also promising. Among 179 planned VBBs reported within the OptiBreech feasibility and pilot studies using this model of care, only one case of serious neonatal morbidity occurred.^8^, ^23^ This is an adverse neonatal outcome rate of 0.6%, compared with 5.0% reported in the Term Breech Trial for planned VBBs.^39^ A similar improvement in outcomes was observed in the evaluation of the physiologic breech birth training program that OptiBreech team members are required to attend, and BSMs are required to teach.^40^, ^41^

The likelihood of greater safety within this model, resulting from the attendance of an experienced practitioner, makes it more acceptable to women who wish to plan a VBB. The standardized pathway also makes the service more accessible. Interviews with service users who received OptiBreech collaborative care within this study also indicated that BSMs were key to successful implementation of a woman‐centered service. That analysis indicated women who wished to plan a VBB had 3 pivotal needs: balanced information, access to skilled breech care, and shared responsibility. The inventory reported in this article reveals some of the processes through which BSMs fulfil these needs.

Women have found OptiBreech care beneficial, regardless of their eventual planned mode of birth.^25^ In a pilot trial comparing OptiBreech care with standard care, women randomized to OptiBreech care had greater access to all care options currently recommended in professional guidance. One in 4 women randomized to OptiBreech care planned a VBB, either before or after ECV, compared with none in standard care (23.5% vs 0%, 95% CI = 9.3%‐37.8%; *P* = .003). Women who requested an ECV also had more successful ECVs performed under OptiBreech care compared with standard care (44% vs 34.8%). At the same time, within OptiBreech care, more women chose a prelabor CB without an ECV attempt (20.6% vs 12.5%). For women with breech presentation, access to both planned VBB and CB without an ECV attempt was articulated as important, due to the pressure often felt to attempt an ECV as the clinician's preferred option within standard care.^25^, ^42^, ^43^

We have described these roles as pertaining to BSMs. Although less common in this study, the roles are also relevant to obstetrician‐gynecologists functioning as breech specialists. Care for women with breech presentation is assumed to be included within all consultant obstetrician‐gynecologists’ scope of practice, but in this research, participants reported that many obstetric colleagues lacked significant experience and confidence to support VBB. This is confirmed by multiple reports on obstetric training opportunities.^9^, ^10^, ^44^, ^45^, ^46^, ^47^ Maintaining the hierarchy requiring obstetric consultants to be solely responsible for all breech care prevents midwives, who have achieved relevant breech competencies, from extending their scope of practice and likely undermines the safety and availability of VBB care. Breech specialists, whether midwives or obstetrician‐gynecologists, enhance access to high‐quality standardized care for women and increase training and support opportunities for colleagues.

### Strengths, Limitations, and Future Work

This thorough description of the BSM role is grounded in rigorous analysis of qualitative data, rather than described in theory, making it directly useful for stakeholders who are most likely to implement the care model or receive care within it. Data obtained using qualitative inventory methodology will enable testing of its replicability at scale. A limitation is that this study encompassed only the first year of implementation of the OptiBreech collaborative care model. Further research is required to assess sustainability and changes in the role as the service embeds within multiple settings. A substantive randomized controlled trial of care within this model is required to confirm improved safety outcomes.

## CONCLUSION

Provision of a safe‐as‐possible breech service requires access to professionals with skill and experience facilitating VBBs, within a multidisciplinary setting. The OptiBreech collaborative care model provides a structured and evidence‐based care pathway for providing a safe and satisfying service for women, with improved training opportunities for professionals. Breech specialist midwives fill important leadership roles within this care model. This research helps to standardize implementation so that it aligns with the improved safety data observed thus far in OptiBreech pilot and feasibility studies.

## CONFLICTS OF INTEREST

SW is a co‐Director of Breech Birth Network, Community Interest Company, a not‐for‐profit social enterprise that delivers vaginal breech birth training and supports research. She receives a standard Director's fee and expenses. Profits from these teaching activities are used to fund further research and teaching activities, such as PPIE expenses, conference attendance, and open access publication fees.

## Supporting information




**Appendix S1**. OptiBreech Physiological Breech Birth Algorithm

**Appendix S2**. Interview Guide: Staff

**Appendix S3**. Extended Collection of Quotes from Interview Transcripts

**Appendix S4**. Standards for Reporting Qualitative Research (SRQR) Checklist
