# Supplementary material for: The OptiBreech Trial Feasibility Study: A Qualitative Inventory of the Roles and Responsibilities of Breech Specialist Midwives
Source: J Midwifery Womens Health. 2025 Feb 1;70(2):270–8. doi: 10.1111/jmwh.13728 (PMC11980765; doi:10.1111/jmwh.13728)
Supplement: Supplementary file 1 — Appendix S1. OptiBreech Physiological Breech Birth Algorithm [file JMWH-70-270-s001.pdf]

Physiological Breech Birth Algorithm

by Shawn Walker RM PhD
Adapted June 2022 from original, published in
Reitter A, Halliday A, Walker S (2020, *Birth*)
with thanks to the OptiBreech Collective

MAXIMUM
7 mins

Steady progress & Reassuring fetal heartrate → **YES** → No interference Quiet, calm, warm support

Rumping
Both buttocks/anus remain visible on the perineum *between contractions*

Delay on perineum → Perineal sweep / buttock lift → Episiotomy → Consider fundal pressure → If pelvis not born immediately following epis & maternal effort – EMCS

Pelvis born → Continuous cyclic pushing → Intervene if pause **>30 seconds** or no/minimal progress with maternal effort AT ANY POINT

< 5 minutes from birth of pelvis

<3 minutes from birth of umbilicus

Incomplete Rotation – *Arm(s) stuck with head, mid-pelvis* → Running start → Sweep down pubic arm

No rotation – *Extended arm(s), pelvis & shoulders remain in A-P diameter* → 180º rotations, release pubic arm

If intervention used, continue to assist until birth complete

Fetal head extended – *at pelvic inlet / not engaged* → **Elevate** *station* & **Rotate** *occiput* → Align – scoop & flex / face-palm

Fetal head deflexed or delayed – *in mid-pelvis or outlet* → Shoulder press / flex head (MSV) → Maternal buttock lift / Suprapubic pressure → Lithotomy / forceps

Birth of the head

Physiological transition

DO NOT cut cord unless HR is <60 bpm and not improving

Evaluate & initiate resuscitative measures at the bedside, **cord intact**
