# Supplementary material for: The OptiBreech Trial Feasibility Study: A Qualitative Inventory of the Roles and Responsibilities of Breech Specialist Midwives
Source: J Midwifery Womens Health. 2025 Feb 1;70(2):270–8. doi: 10.1111/jmwh.13728 (PMC11980765; doi:10.1111/jmwh.13728)
Supplement: Supplementary file 2 — Appendix S2. Interview Guide: Staff [file JMWH-70-270-s004.docx]

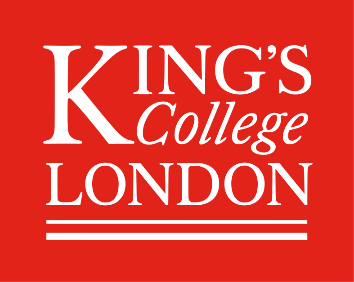


**Interview Guide: Staff**

The first guide is for sites in which a breech team has been formed and attended at least one vaginal breech birth during OptiBreech 1. The second is for sites where vaginal breech births have not occurred and/or a breech team has not been formed during OptiBreech 1.

**Guide 1**

| **Focus** | **Breech Team Members** | **Other Staff** |
| --- | --- | --- |
| Background | Tell me about your role in your perinatal service. | Tell me about your role in your perinatal service. |
|  | Tell me about your experience with breech birth in your career, prior to this research. | Tell me about your experience with breech birth in your career, prior to this research. |
|  | How did you become involved with the breech team? | How have you been involved with the breech team during this research? |
| Affective attitude  *How an individual feels about an intervention* | How do you feel about the breech team?  If they talk about confidence in a particular person: What about [name] gives you confidence? | How do you feel about the breech team?  If they talk about confidence in a particular person: What about [name] gives you confidence? Or why do you not feel confident in the team or [name]? |
| *Burden*  *The perceived amount of effort that is required to participate in an intervention.* | Tell me about what participation in the breech team has required of you. | Tell me about what your hospital’s provision of a breech team has required of you. |
|  | How have you had to adjust your usual practice? | How have you had to adjust your usual practice? |
|  | How did the breech team service build on your previous skills and experience? Or was it completely new? |  |
| Self-efficacy  *The participant’s confidence that they can perform the behaviours required to participate in the intervention.* | Tell me about your level of confidence around your role in the delivery of the breech team service. | *Clinicians* – Same; or  *Managers* – Tell me about your level of confidence in your team’s ability to deliver the breech team service. |
| Ethicality  *The extent to which the intervention has good fit with an individual’s value system.* | What is important in the provision of a ‘good’ breech service? | What is important in the provision of a ‘good’ breech service? |
| Intervention coherence  *The extent to which an individual understands an intervention and how it works.* | Describe to me how the breech team service works in your hospital. | Describe to me the standard care pathway for women with a breech pregnancy at term in your hospital, prior to the implementation of the breech team. |
|  | How does this differ from standard care provided to people with a breech-presenting fetus at term? | How has the breech team changed things? |
|  | What is the breech team meant to achieve? | What is the breech team meant to achieve? |
| Ethicality  *The extent to which the intervention has good fit with an individual’s value system.* | How do these differences affect your service’s ability to provide ‘good’ breech care? | How do these differences affect your service’s ability to provide ‘good’ breech care? |
| Perceived effectiveness  *The extent to which the intervention is perceived to be likely to achieve its purpose.* | In your view, how does the breech team affect pregnant people’s ability to access a vaginal breech birth? | In your view, how does the breech team affect pregnant people’s ability to access a vaginal breech birth? |
|  | In your view, how does the breech team affect training opportunities for perinatal care staff members? | In your view, how does the breech team affect training opportunities for perinatal care staff members? |
| Opportunity costs  *The extent to which benefits, profits or values must be given up to engage in the intervention.* | Describe to me any changes that may have occurred as a result of the breech team that you were not expecting. | Describe to me any changes that you have observed may have resulted from the breech team that you, or perhaps the research team, were not expecting. |
|  | Describe to me what you feel it has cost the service to provide the breech team. | Describe to me what you feel it has cost the service to provide the breech team. |
| Summary | Would you recommend a breech birth in your hospital to a friend or family member? | Would you recommend a breech birth in your hospital to a friend or family member? |
|  | Is there anything else you would like to tell me? | Is there anything else you would like to tell me? |

**The OptiBreech Intervention Logic Model**, Sept 2021

**Things for the interviewer to consider in order for the data to help refine the current Logic Model:**

Needs

- Does the person feel that women are able to choose to give birth vaginally to their babies within their Trust? And how does the OptiBreech team effect this?
- Does the person feel that care from the OptiBreech team is reasonably low-risk for women who plan a vaginal breech birth? And how does this compare to standard care?
- How does the person feel the OptiBreech team impacts births where breech presentation is diagnosed for the first time in labour?
- What are the potential drawbacks of having an OptiBreech team?

Context

- Does the person feel their exposure to vaginal breech birth has increased due to the OptiBreech team? How do they feel about this? (Good? Bad? Bit of both?)
- How does the person feel about their training opportunities around vaginal breech birth?

OptiBreech Intervention Inputs

- How does the person feel about the proficiency criteria? (overall 10 vaginal breech births, 3 per year, completion of physiological breech birth training, delivery of training and reflexive reviews)
- How does the person feel about the role of the Breech Specialist Midwife? What are the potential drawbacks or barriers for this role?
- For managers: How should the Breech Specialist Midwife post be funded?
- How does the person understand and feel about the ‘physiological breech birth’ approach? What aspects of the intervention influence these feelings? The Algorithm, time limit recommendations, upright maternal birth positions, manoeuvres specific to upright birth?
- For PIs/Breech Lead Midwife / Breech Lead Obstetrician especially, but all interviewees: How does the person feel about the mentorship available through the research team and Breech Birth Network training and webinar activities? What have they participated in? What has changed about their practice? What has been most/least helpful?

Context

- How does the person feel about the dedicated Breech Clinic? (whether there is one, or is not one) How do they feel this influences care, or how might it?

Outputs

- How does the person feel mandatory training activities have changed as a result of the activities of the OptiBreech Team or the Breech Specialist Midwife?
- How does the Clinic and/or OptiBreech team effect the training of obstetric registrars and midwives?
- Has the person experienced any reflective reviews of OptiBreech births? If so, how did they feel about it?

Short term outcomes

- Does the person feel more or less confident, or no different, to offer vaginal breech birth to a woman, depending on whether her birth will be supported by the OptiBreech team?
- Does the person feel more or less confident, or no different, to attend a vaginal breech birth if an OptiBreech Team member is present? The Breech Specialist Midwife? The Breech Lead Obstetrician?
- Does the person feel confident that all of their obstetric consultant colleagues are happy and able to support women who choose to plan a vaginal breech birth?
- What is the persons perception of how the OptiBreech team and/or the Specialist Midwife has affected outcomes of vaginal breech births?

Medium term outcomes

- Does the person feel vaginal breech births happen regularly in their setting? Has this changed?
- How reliant is the service on one person? Is there an on-call system? Do others feel confident to support these births?
- How satisfied is the person overall with the way the OptiBreech team operates in their hospital?

Longer term outcomes

- Does the person feel they have acquired skills they can take with them to another setting, where there may not be a Specialist Midwife, Breech Lead Obstetrician or OptiBreech team.

**Guide 2**

Gathering data in sites where physiological breech births have NOT occurred and/or a breech team has not been successfully formed is particularly important. This will enable a fully understanding of why the model may be successful in some sites but not others, and what the main barriers and facilitators are, especially through comparison with sites where the model has worked. This understanding will help to refine the OptiBreech programme theory and Logic model (p25) so that it includes more information about context.

Interviewees will include the local PIs and other key stakeholders they identify who consent to participating in an interview.

| **Focus** | **OptiBreech Team Members** |
| --- | --- |
| Background | Tell me about your role in your perinatal service. |
|  | Tell me about your experience with breech birth in your career, prior to this research. |
|  | How did you become involved with this research project? |
|  | Tell me about the process of attempting to provide a breech team as part of this research in your hospital. |
| Affective attitude  *How an individual feels about an intervention* | How do you feel about the breech team as described? |
|  | Why do you feel it has not worked in your hospital? |
| Intervention coherence  *The extent to which an individual understands an intervention and how it works.* | As you understand it, what is the breech team meant to achieve? |
|  | How does this differ from standard care provided to people with a breech-presenting fetus at term? |
| Burden  *The perceived amount of effort that is required to participate in an intervention.* | Tell me about what participation in this research has required of you. |
| Self-efficacy  The participant’s confidence that they can perform the behaviours required to participate in the intervention. | Tell me about your level of confidence around your role in the delivery of the breech team. |
| Ethicality  *The extent to which the intervention has good fit with an individual’s value system.* | What do you feel is important in the provision of a ‘good’ breech service? |
|  | What have been the main concerns around implementing the breech team? – I am interested in your concerns, as well as what you understand of your colleagues’ concerns. |
| Perceived effectiveness  *The extent to which the intervention is perceived to be likely to achieve its purpose.* | How might the breech team affect pregnant people’s ability to access a vaginal breech birth? – I am interested in your view, as well as what you understand of your colleagues’ views. |
|  | In your view, how might the breech team affect training opportunities for perinatal care staff members? – I am interested in your view, as well as what you understand of your colleagues’ views. |
| Opportunity costs  *The extent to which benefits, profits or values must be given up to engage in the intervention.* | Describe to me what you feel it would cost the service to provide the breech team. – I am interested in your view, as well as what you understand of your colleagues’ views. |
| Summary | Is there anything else you would like to tell me about the barriers you have encountered when attempting to implement the breech team? |
