# Supplementary material for: The OptiBreech Trial Feasibility Study: A Qualitative Inventory of the Roles and Responsibilities of Breech Specialist Midwives
Source: J Midwifery Womens Health. 2025 Feb 1;70(2):270–8. doi: 10.1111/jmwh.13728 (PMC11980765; doi:10.1111/jmwh.13728)
Supplement: Supplementary file 3 — Appendix S3. Extended Collection of Quotes from Interview Transcripts [file JMWH-70-270-s003.docx]

**Appendix 2.** Extended Collection of Quotes from Interview Transcripts

| 1. **Care Coordination and Planning** | |
| --- | --- |
| **1.1 Coordinating the Breech Clinic** | *But I am increasingly getting referrals from other people as well. Some doctors, mostly midwives who encounter women and say can you contact this person.* (S100, midwife, specialist)  *So I have set up the clinic, although I do have a dedicated consultant cover for that, but I manage all of the referrals, have all of the conversations with the women.* (S101, midwife, specialist)  *For me personally, it's one of the favourite aspects of my job because I get to run my own independent clinic.* (S101, midwife, specialist)  *I am constantly prompting the rest of the team to come and do some teaching with me and cover the clinic when I'm away. And so that is getting better now, I've got a couple of people that are going to cover the clinic when I'm coming up.* (S101, midwife, specialist)  *… then the plan sort of for the rest of the flow -- for the rest of the service -- is to actually sort of set up, we're going to call it the OptiBreech Clinic, where women there will be a referral system that comes through electronically.* (S102, midwife, specialist)  *… it'll probably be about two a week on average for a service our size so they will be counselled by me and then we'll run like an ECV OptiBreech Clinic out of our birth centre.* (S102, midwife, specialist)  *It's being able to operationalise the clinic, so that's about space, it's about people that are gonna staff it, how that fits into their-- into the wider operational plan of the workforce.* (S102, midwife, specialist)  *I think that it needs to have a clinic where women are referred into.* (S104, midwife, specialist)  *So, yeah, the hope is going forward, we'll have a midwife-led breech clinic. With a doctor working alongside.* (S105, midwife, specialist)  *I've taken over their care completely from the point at which they're diagnosed.* (S105, midwife, specialist)  *This is actually now the proper breech clinic, and looking after all the breech patients is [OptiBreech staff].* (S106, midwife, colleague)  *My ideal would be to have a breech clinic, because at the moment ECVs are just done on random days where a woman could come, have her options discussed, meet one of the breech team midwives who would be potentially able to come and facilitate second stage whilst having routine care in labour.* (S108, obstetrician, specialist)  *The time and implementing clinic, you know, if you're going to take up a new clinic that takes time and money, of course, and the venue.* (S109, midwife, colleague)  *So, the consultant midwife is the lead person who we’re trying, they’ll try and refer her them through to here because she has some capacity in a clinic fashion.* (S115, midwife, colleague)  *I think the clinic is gonna be the biggest aspect. I think once we get a clinic up and running, because that's a lot of what I spend my time doing is trying to contact women, arrange when we can meet and and when it tends to be on Dave’s roster, so days that he's already working, it's trying to juggle these women amongst Dave's work already. So if I've got, you know, I've got a clinic and I could just arrange women to see them there.* (S116, midwife, specialist)  *She has a clinic of women that she sees, and then equally if we have ladies that come in for ECV… so our ECVs are performed on the labour ward at [S117]. If they’re either unsuccessful or actually when they arrive we’re not, the ladies maybe don’t engage, aren’t as engaged with the process as we’d like them to be, then we offer them Jess’s details, she contacts them straight away* (S117, midwife, colleague)  *Erm, the ladies all know her, she is a very active part in their pregnancies and attends the clinic.* (S117, midwife, colleague) |
| **1.2 Managing the Breech Team** | *I also have access to the midwives on call list and the labour ward coordinators. So I know when someone who meets the criteria are working. So I just make sure that there's always someone on. And I just do that a few days in advance.* (S100, midwife, specialist)  *So I've got different people interested in offering different things within the service, so it's just trying to put all those together so that we can actually create that cover.* (S102, midwife, specialist)  *There's not gonna be any on-call element to it. So it is going to be very much whether they're on shift at the time.* (S103, midwife, specialist)  *Um, so the plan will be that when a woman says that she wants to have a vaginal breech birth, we will put together an on-call rota to cover. And it might be some of the midwives are shift based anyway, so if she comes in, if they're on shift, they'll take her. And obviously, if not, we'll call something from home.* (S104, midwife, specialist)  *So I mean at the moment what I'm trying to do, I have to look at their existing off duty after it's been done and write down in which shift, which member of the breech team is available for that shift.* (S105, midwife, specialist)  *So what I've tried to do, the labour ward manager who does the off duty, I have said to her, “Look, we need these midwives to be on different shifts. They can't be on the same shift.” And I want them really, ideally on a different shift to when one of the breech consultants is on a shift just so they can spread out a little bit and have greater cover. This hasn't happened, and I'm not sure why it's not happened. So that's another reason why I'm going to meet with the lead intrapartum midwife and to kind of address that issue.* (S105, midwife, specialist)  *And so there's a lot of communication with each other that is going on that I feel is, it's just feeling like a bit more of a team effort rather than me doing something new in the hospital.* (S100 midwife, specialist)  *I think, as a team, what we have already created is absolutely excellent. You know, we have a WhatsApp group and we support each other, and this is amazing* (S105, midwife, specialist)  *I've also done in one of our breech meetings I did a kind of reflection on that birth just so the other members of the team could kind of learn from that birth as well.* (S105, midwife, specialist)  *I've told them they need to come to one in three meetings just to keep their skills and their knowledge up to date and, you know, to share learning and things like that. So my role as a leader has kind of developed, I suppose, and I'm learning a lot about leading a team through doing this.* (S105, midwife, specialist)  *[OptiBreech staff], she's available constantly for us, and she supports us so brilliantly.* (S106, midwife, colleague)  *…having that breech team and having the support can actually develop much more, can actually inspire much more people and develop in the much more the maternity unit.* (S106, midwife, colleague)  *So I think I've kind of mentioned the support, having one person having oversight over all women, and having regular communication between members of the team and regular meetings for reflection. I think those the main things I think are really important to keep that service going.* (S109, midwife, colleague)  *… we can talk about the experiences we’ve had, what we can learn from the experiences that we had, how we can improve our practice. So yes, I think the OptiBreech team is coming along nicely.* (S109, midwife, colleague)  *But we have regular OptiBreech meetings and we have an OptiBreech Whatsapp group, which is multidisciplinary as well. So we have obstetricians, midwives, support workers that are part of OptiBreech team. So we can communicate easily and effectively on that as well.* (S109, midwife, colleague)  *And I know that I've also got good support with the breech team as well if I need it, yeah I’d say I’m relatively competent and comfortable with it.* (S112, midwife, colleague)  *… she's always said to me that she's very happy for me to contact her and discuss any case, any situation I can call her whenever if somebody comes in, and I think something's been done wrong.* (S112, midwife, colleague)  *I think, again, having the consultant midwife there or available? Or indeed, like, just the presence of her in the in the, the team in the senior team does make a difference to people's confidence.* (S113 midwife, colleague)  *… we're trying to set up some regular training meetings which you know, obviously will be open to other members of staff…. Build a community of practice and have that support for each other.* (S114, obstetrician, specialist)  *So we got to the point where we've got our team, we've got a WhatsApp group, we're all talking to each other. We've had an initial meeting, we're setting up some training in terms of more regular kind of chats amongst ourselves.* (S114 obstetrician, specialist)  *And that's where the team is helpful because you're hopefully all saying the same thing because we're all trained in the same way and we're all coming from the same viewpoint. So, you know, I mentioned about jobs for emergency section. So if we can get, make sure that a woman who comes in with a breech in labour, whether it's known about in advance or undiagnosed, can see one of the breech team so that we're consistently saying the right thing and you've not got that kind of throw of the dice of who you happen to see on that day.* (S114, obstetrician, specialist)  *Then, because I've got a lot more confident, I had a lady come in when I was on call over Christmas, and I felt confident enough to do a physiological breech birth with her. Which there was, like one point, I needed to do something and I did feel a bit like, “Oh, my goodness, did I do the right things,” but I did a little kind of reflection and debrief with [OptiBreech staff].* (S114, obstetrician, specialist)  *I think the thing that makes different as well in terms of the whole, the OptiBreech team is being able to have regular meetings so we can discuss and refresh, especially where there's periods where you don't have any referrals for people to have a plan to have a vaginal breech birth so there isn't a need for any on calls. Erm, I think it's really beneficial for people to come back and have a chat and discuss and also to go through like a little bit of training or like simulation or demos and that sort of thing just to, be able to discuss it.* (S115 midwife, colleague)  *What's really lovely and really enjoyable is that we've got obstetricians and midwives involved, and when we have team meetings, it's very interesting to have the discussion between the midwives and obstetricians and the different experiences, different comfort zones, and being able to have those sort of like open discussions and it's really positive to have obstetricians on board who are supportive of breech because so often the colleagues cannot be. Even, and what’s really nice as well is the obstetricians who are involved are, will sometimes admit to or be very open and honest about areas of discomforts with you with breech, which is really nice to be able to have those kind of relationships.* (S115, midwife, colleague)  *So one of our consultants who's on the team is our clinical director. Erm, and we both attended a breech birth together, and it was really nice to know there was somebody else there in the room who was OptiBreech as well as just me, so it wasn't all on me…. and to see how excited she was about what had just happened, was really nice about to like share a little bit of, you know, “We're both excited about it, we've just had a successful vaginal breech birth and that was really nice, and that's really exciting and really positive,” which I think gives you a little bit of confidence that someone else is on board with you and has got your back and able to support you, if things are difficult. Yeah.* (S115 midwife, colleague)  *Either myself or the consultant will put together the on call rota based on people's current working patterns, and then if there's just aren't covered, we'll put it out to the group and say, is anyone happy to cover any of these on calls? What we started to do with that on call rota as well as put a first and a second on call.* (S115, midwife, colleague) |
| **1.3 Individualised Care Planning** | *I find that once you start to provide consistent support and a pathway that is easy to access, you have a much bigger range of women… And that's why I think that having a dedicated care pathway where someone doesn't actually have to call out the army in order to arrange appropriate support makes it more equitable and just.* (S100, midwife, specialist)  *And I had to just keep playing the broken record and saying, “She's made her informed decision, I've counselled her about what impact that could have on her birth. She's made an informed decision and this is the plan. I'm not changing the plan anymore because -- for a non-indication.”* (S100 midwife, specialist)  *So it's predominantly led by me, but I get an obstetrician involved just to make sure that it's a collaborative care plan, really.* (S101 midwife, specialist)  *I've got quite an extensive counselling form for women that are having a vaginal breech birth, and I make sure that that's all signed and uploaded onto all of the systems and everything so that people feel safe.* (S101, midwife, specialist)  *…. there will be some members of the obstetric and midwifery team which don't feel confident at all, or actually are very against women having these choices. And so therefore it requires quite a lot of personal strength to be able to navigate that. So I think sometimes, in a couple of cases -- one lady that didn't have a vaginal birth -- you know, various minor complications were adding up, but the support from the wider team was really lost, and I felt quite a lot of pressure to navigate between the women and the wider team.* (S101, midwife, specialist)  *So all women were getting the same information and they were being counselled in the same way. But being able to individualise that, depending on other complexities that the women may have had in their pregnancy or medical history.* (S103, midwife, specialist)  *...and then she's just creating the birth plan for the for the breech delivery.* (S106, midwife, colleague)  *And then she is, we have the folder so if there's any lady who decides to have a breech delivery, we have got all the information in the breech folder.* (S106, midwife, colleague)  *I went down to delivery suite and said right, what’s changed and they said right here’s the folder um, and it really was all the Optibreech information for trial participants you know, so they now know that, you know, if we get a lady who’s coming in who’s agreed to participate or whatever. This is the process that we follow and all the proforma are in the folder and who needs to be notified, and obviously these ladies have got their birth plans and their birth plans are posted and shared and the designated midwife is identified, who’s to be called when this women presents in labour.* (S107, midwife, colleague)  *And supporting them with creating personalised care planning. Particularly for the women that choose to go to the birth centre or plan to give birth at home with a vaginal breech, having a vaginal breech birth at home and ensuring that they are following the pathways and referring to [OptiBreech staff] and that the right care has been offered recommended implemented.* (S109, midwife, colleague) |
| **2.0 Service development** | |
| **2.1 Writing Policy and Guidelines** | *I felt that we definitely needed to get the policies and processes in place first, so I needed to get agreement for the change in management for vaginal breech [inaudible] approach before I started operating that on the clinical floor, because now I can reassure people that actually, “No, you're supported in the policy by this”. And prior to me joining, only an obstetrician could deliver a breech baby and it had to be delivered in theatre. And so now I can support the midwives and say, “Actually, that's not the case anymore, and the policy supports that”.* (S101, midwife, specialist)  *And I was then in a position where I could have that influence over the education and I could actually go back and say, Right we're missing a trick here. We're only offering half a set of options, we're only trained in one way we've got a lack of understanding. So from that, I was able to change the guidelines, change the education, roll it out.* (S102, midwife, specialist)  *So yeah, so my focus is around counselling women who want to give birth outside of guidance, and in writing new guidelines, updating guidelines and getting involved in research to bring to the trust.* (S104, midwife, specialist)  *And in the first meeting, I said to her, We need a new guideline first of all, because the old guideline was very outdated. And she said, Well, why don't you write one? And so it went from there. So within the guideline, I put the new pathway and how the breech team would run, and that obviously had to go through governance and all sorts and lots and lots of, you know, tweaking and everything. And I had had, you know, lots of people kind of reviewed it before it got released.* (S105, midwife, specialist)  *Once I started, I was allowed to write new guidelines. So I did that and I recruited the breech team. So we had a new pathway in the guideline as well that I wrote.* (S105, midwife, specialist)  *I know [OptiBreech staff] was doing an update to guideline.* (S114, obstetrician, specialist)  *Our guidelines are in continuous development. I mean they they still going on and we find new things as we go and we will keep finding new things and new methods and new, uh new probably results or new evidence after this, and that's what we want to know whether what we're doing is good enough or we can do more, or we should do less. And this is the only way forward. We have to write everything down and then and then see.* (S116, obstetrician, specialist) |
| **2.2 Standardising Information and Referral Pathways** | *Whereas I find that once you start to provide consistent support and a pathway that is easy to access, you have a much bigger range of women… And that's why I think that having a dedicated care pathway where someone doesn't actually have to call out the army in order to arrange appropriate support makes it more equitable and just.* (S100, midwife, specialist)  *I think it's made it much easier because nearly everyone who is breech goes through that clinic…. But I am increasingly getting referrals from other people as well. Some doctors, mostly midwives who encounter women and say can you contact this person. But the clinic does make research a lot easier because not everyone who is known to be breech passes through.* (S100, midwife, specialist)  *So the problem with recruiting, and this has been shown in previous studies with breech studies is that if you don't have a breech clinic care pathway, women will come through to so many antenatal clinics. And you almost don't know which ones are breech because it's not necessarily recorded that that's why they're going to the clinic. And so they're hard to come across. So they talk to 50 different people. Whereas at a breech clinic service, they're at least talking to one person.* (S100, midwife, specialist)  *But we have a separate pro forma. And I go through that pro forma with people.* (S100, midwife, specialist)  *… women used to just see whoever they wanted and have very varied experiences of what information was provided to them and what options were available to them.* (S101, midwife, specialist)  *I've got quite an extensive counselling form for women that are having a vaginal breech birth, and I make sure that that's all signed and uploaded onto all of the systems and everything so that people feel safe.* (S101, midwife, specialist)  *An area that I really tried to work with is sonography. So I've had a couple of meetings with the sonographers, and I've shared with them the policy and the pathway, because I definitely felt that was an area that might have been missing out on the information. And actually, sonographers are often the first people to diagnose a breech birth, and if they straightaway say, Oh, you're gonna have to have a caesarean now, that fear or that seed is already sowed. So I've really tried to work with the sonographers to say, “Just refer the women to the clinic”, so the sonographers directly refer women to us now. So that's helped quite a lot.* (S101, midwife, specialist)  *Thinking about consultants, it's about -- at the moment the ECVs are just brought into Labour Ward and whoever's on the Labour Ward does the ECV, for example, and then they get re-counselled if it doesn't work, if it does work. So any consultant or any registrar could be providing that. And that's where the discrepancies in counselling come in.* (S102, midwife, specialist).  *So the woman presents breech, either suspected on palpation or confirmed on scan either coincidentally or just confirmed by presentation scan, that referral comes through to me, and then they're counselled by me … But what that will enable us to do is to try to standardise the counselling that they get antenatal about their options so that they can make the best choice for them.* (S102, midwife, specialist)  *And that's why I just need to get the women coming to me as the first line and not seeing a junior doctor that's petrified that if the baby comes out breech, something awful is going to happen every time.* (S102, midwife, specialist)  *… we have, probably some biases in how women are counselled. It's not very standardised, it's not -- it depends on who you get and what their personal experiences of breech are to how they counsel women around their choices.* (S102, midwife, specialist)  *… but actually, one of my bigger challenges is getting access to these women in the first place. So, actually, knowing how many women are coming through and presenting breech after 36 weeks. So that I can actually get in touch with them and identify the people that would be eligible to be cared for by the breech team. So you've got your operational side of it, and then you've got the actual women's side of it, so they're both really important.* (S102, midwife, specialist)  *… there will be a referral system that comes through electronically. So the woman presents breech, either suspected on palpation or confirmed on scan either coincidentally or just confirmed by presentation scan, that referral comes through to me.* (S102, midwife, specialist)  *They get really nice standardised counselling about their options.* (S102, midwife, specialist)  *I mean, it's not really an ethical service because not everyone is getting the same counselling. So not everybody is being provided with all the options.* (S103, midwife, specialist)  *All women that had a breech presentation were referred in to see me, and I was able to counsel them in all their options.* (S103, midwife, specialist)  *It seemed like the best way to do it because it meant that everyone was getting the same information. So all women were getting the same information and they were being counselled in the same way.* (S103, midwife, specialist)  *It's an obstetrician. So it's not a member of the breech team. It might be, if one of those obstetricians there but it could be any obstetrician because it just depends who's there at the time. So that's an issue.* (S105, midwife, specialist)  *So when a woman has a breech suspected at 34 weeks, then the community midwife will give her an information leaflet.. So it just basically says you will have a, if baby stays breech, you'll have the option of an ECV and if it's not successful or you choose not to have an ECV, then you've got the choice of having a vaginal breech birth or Caesarean section. But that is very basic at that stage. At 36 weeks, if women have a suspected breech presentation, they would then get referred for a presentation scan. And obviously if it was diagnosed as breech presentation at that scan, they would then remain in the antenatal clinic to be seen by one of the doctors, one of the obstetricians. What the obstetricians are then meant to do, are offer an ECV and counsel them for an ECV, that they're meant to give them printed information, which is in our guidelines.* (S105, midwife, specialist)  *I think the difficulty with recruiting women at the moment is that because we've got a new pathway, nobody is following it at the moment. And I think if it was followed, I think we'd recruit more women. So that's another issue that I need to address really. So I have emailed the lead labour ward consultant just to say, this pathway is not being followed and really we need a few more breech births in the next few months just to, you know, increase the team's confidence before I go on leave.* (S105, midwife, specialist)  *The only thing that I mean, the women that I've cared for have commented on is the fact that they have had continuity. So because it's been me that's had to be on call for these women personally, I've taken over their care completely from from the point at which they're diagnosed. So I've done the antenatal visits just to try and build up a relationship with them so that, you know, when I'm looking after them in labour, they they've got more of a trust in me. And then obviously I can't then just leave them after they've given birth so I'll do the postnatal care as well. And again that, having a specialist role would enable you know me to carry on doing that, you know, for all the women, and I mean, I'm a continuity midwife anyway, so it's what I do and I think that's been really important. We know that continuity of care does achieve better outcomes. So that's kind of part of the reason as well that I wanted to kind of properly care for these women not just say, “Oh I'll be there at the birth and that's it.” And I think it's important that when we move forward and the breech team is actually working better, that these women perhaps have a chance to meet some of the members of the breech team or at least be able to to know who they are, so that at least they know a little bit about them, you know, when they're looked after by them in labour. So I think that continuity is an important factor as well.* (S105, midwife, specialist)  *I think a lot of women would probably be counselled against a vaginal breech, certainly by the registrars on the day assessment unit, not -- potentially by the consultants who do the ECVs.* (S108, obstetrician, specialist)  *And I think there will be more involvement for the optibreech team to be involved in kind of ECV clinics and discussion and options and things*. (108, obstetrician, specialist)  *…but also that in the future, when this is kind of a very established thing in, in the trust, that will be improving women’s outcomes by having that continuity and expertise and knowledge. And yeah, so having an impact on their outcomes essentially.* (S109, midwife, colleague)  *… I think often because people have read stuff on the internet, they come in expecting that caesarean is going to be the only option and then are surprised by the choice of physiological breech.*  (S113, midwife, colleague)  *But normally, I think that's been one of the roles of consult midwife to kind of scoop people up and to give them the full range of information.*  (S113, midwife, colleague)  *I've had people who are presenting breech and have spent time with a consultant midwife making decisions. And again, it's so it's been so valuable to have someone that they can go to, I appreciate that one person can't cover everyone. So, the more that that knowledge can be filtered down, and the birth options clinic has some of those skills as well, to really, you know, present all the options.* (S113, midwife, colleague)  *… one of the problems we have is people giving conflicting information.* (S114, obstetrician, specialist)  *Yes, I do. I think it was a bit more ad hoc, a bit more potluck about who you might see before. Whereas now, what we're trying is if the sonographers pick someone up as breech, they refer straight to being hopefully randomized, and then, you know, whether [OptiBreech staff]'s having a session with them.* (S114, obstetrician, specialist)  *It's people writing the wrong things down or being ignorant and not understanding. So I think that consistency is really important.* (S114, obstetrician, specialist)  *And that's where the team is helpful because you're hopefully all saying the same thing because we're all trained in the same way and we're all coming from the same viewpoint. So, you know, I mentioned about jobs for emergency section. So if we can get, make sure that a woman who comes in with a breech in labour, whether it's known about in advance or undiagnosed, can see one of the breech team so that we're consistently saying the right thing and you've not got that kind of throw of the dice of who you happen to see on that day.* (S114, obstetrician, specialist)  *So we have told our colleagues that we have a breech birth trial going on and we would like them to refer the cases to us to counsel. Now a lot of our colleagues are referring women with breech babies. Sorry. And when they when they are happy to have that counselling, we usually do them in our own time, especially [BSM name redacted].* (S116, midwife, specialist)  *I think it's just that familiarity with the member of staff and just feeling more comfortable and knowing, knowing what’s already been discussed and maybe they've got further questions from previous discussions and that seems to be much better to be found as we've had positive feedback regarding that.* (S116, midwife, specialist)  *And I think if women are aware and empowered, they can then ask for things and but you know, regardless, and we're in the trial information out there, it's the word is out in the community, there's lots of community posting, and word of mouth from what I hear.* (S118, obstetrician, specialist) |
| **2.3 Promoting Cultural Change** | *And I sometimes feel a bit sad about that because I feel like I talked her out of breech birth almost for political reasons, so that everybody didn't see me as someone who was reckless when it didn't really have anything to do with me. And so sometimes I feel like I have to think too much about whether others will perceive me as a reason why women are willing to take these risks, as what they perceive as risks. Just because I'm supporting informed decision.* (S100, midwife, specialist)  *…so one thing that has been challenging is that some of the midwives still find it challenging to see me as an expert, rather than feeling that I'm -- that the obstetricians are the experts.* (S100, midwife, specialist)  *I didn't feel supported by her, which was really challenging because I'd go out of my way to tell everyone that she is the expert on external cephalic version, and say it in front of her, say it when she's not around and really honour her expertise. So I would really like to feel that coming back, but it's not 100% yet.* (S100, midwife, specialist)  *When you get down to band 7, I think there are a few doubters. And I try to talk to people personally and give them time to ask questions and to explore things, but also without putting anyone on the spot. Because sometimes people just need time to warm up.* (S100, midwife, specialist)  *Yeah, I think so. I think that it can take quite a while to change. And one of the other factors that is going to be influential quite soon is that the doctors will change hospitals. Every August, doctors will rotate from hospital to hospital. So again you'll have a new batch of doctors and a new opportunity to teach, to learn. And so we'll see what happens when that changes. But yeah, I think that I can already see it in the people just on the WhatsApp group that they're becoming more confident about seeing themselves as people who will teach other staff. Which I think is one of the really important parts of the breech team. Because it's not just about a team of people who attend the breech births, it's about a team of people who are leading the service and are seen as the experts in care for that sub-population.* (S100, midwife, specialist)  *Those people in the team feel confident to support vaginal birth with support, and they are also the spokes across the service to be the little beacons of advice and positivity around vaginal breech births. So I think that we are slowly infiltrating the traditional norm.* (S101, midwife, specialist)  *So, mostly the doctors that I've engaged has been our consultant group because I generally find you get your consultants on board the rest seem to follow. They've been, so before we enrolled in the trial I took it to the consultant meeting, and the consensus was, “Yes this is something we can do,” which is great.* (S102, midwife, specialist)  *My previous Trust, I had been there for eight years, so I already had established relationships with the obstetric team and very good working relationships through my role that I had there anyway. And so it was much more easier to set up the service, because I had built that trust and that rapport up with the wider team and they knew what my clinical skills were, and they knew how that -- I think they knew my philosophy, they knew how I counselled women. So it was very much easier to be able to go to an obstetrician and say, you know, “This is what I want, this is what I've said to the woman.” And them to be able to trust that.* (S103 midwife, specialist)  *Whereas now here at [site redacted], I'm having to build those relationships and it's a very different unit. It's -- clinically, the clinical decision making team are very different, although they are probably more supportive of breech births from a from a starting point and say that my previous Trust was before. But building those relationships and trying to get people on board has been more difficult*. (S103, midwife, specialist)  *I think what I would like to see is obstetricians being a bit more forthcoming about maybe recognising that, and attending training, and attending births so that they can see the way that I practise, it works, the manoeuvres that we use, they work.* (S103, midwife, specialist)  *And so what we've also done is we've just had our new intake of doctors. So we put on the physiological breech training afternoon for them to kind of go through it. So we're just going to try and bring it in that way, of doing kind of regular skills and things, and addressing consultant concerns separately because I think they probably wouldn't want to speak out quite so much in front of junior doctors. So, yeah, we're kind of taking it from providing lots of training and information for them.* (S104, midwife, specialist)  *… we are putting together a presentation to go to the consultant audit meeting in, later on this month, where the PDM [Agata] will be presenting about it so that they all feel aware. And I don't think it's so much that they're opposed to it. I just think they feel a bit like, because they're not leading on it, that it's been, you know, taken out of their hands or something. So I think a few of them just have the nose out of joint a bit, but not necessarily against it, just yeah, letting their feelings be known.* (S104, midwife, specialist)  *I was a bit worried about how the consultants would view it, but I was in a good position before I started in that I'd already done a year of teaching breech which many of the consultants had been to.* (S105, midwife, specialist)  *Because I don't think they're taking it seriously to be honest. Because I'm not in the specialist role, it's just like being run by a band six midwife, which is is mad because, you know, the breech team are band seven midwives and consultant obstetricians. So yeah, I don't know* (S105, midwife, specialist)  *I suppose it's, in terms of, like, networking and you know, getting to know other professionals and them getting to know me that's a massive thing that's happened. Obviously everybody knows my name now, and goes, “Oh, you're the breech midwife.” You know, even if they haven't met me before, face to face, I'll get consultants going, “Oh you're, that's you who does the breech birth.” So you know, that's been a good thing, to kind of get that respect in a way, because I was really just unknown before I started doing these births. Because people on the unit wouldn't see me very much, because I was, I'm based out in the community a lot. So that's a big thing and, you know, multidisciplinary working, working with the consultants, and I was very nervous when I started doing the meetings with the consultants and things like that. It's, you know, as a Band 6 midwife, it's a difficult thing to kind of do and kind of, you know, lead on, but as I've done more and more, you know, my confidence has grown because actually all the questions that I faced from the consultants, I was able to kind of give them an answer. So that made me feel a bit more confident in my own knowledge as well.* (S105 midwife, specialist)  *We need to inform people we need to educate them. They need to know that is not dangerous that we can do it. I think informing people educating them giving them the right training is going to give them confidence and then eventually there will be more people wanting to join the team or simply we're not probably not going to need a team. Maybe in the future, we're just going to do it as a trust.* (S110, midwife, colleague)  *I think over the time, it's been very much like a taboo going for a breech vaginal breech birth. And so now, it's we've gotten away from the whole breech is a version of normal. And everybody's kind of scared of it. And I think it's needed to have the extra training for staff and, and then being able to see more breeches, and then everybody else feeling more confident, because they see more breeches. And so it kind of has a knock on effect.* (S112, midwife, colleague)  *I feel like there's a there's a feeling of positivity around it. And a kind of humility as well. I mean, everyone's aware that you know, it's you don't know what you're doing unless you've had more experience and that we will always be leaning on those who know more.* (S113, midwife, colleague)  *I think, again, having the consultant midwife there or available, or indeed, like, just the presence of her in the in the, the team, in the senior team does make a difference to people's confidence.* (S113, midwife, colleague)  *I mean, certainly the breech service has been enhanced and everyone knows within the trust that we have a specialist and that she is flexible and you know, willing to be on call both in service at the families but also in services, the research and that she's been involved in, you know, developing with [OptiBreech staff], the algorithm and other things that make a big difference to our understanding of what's safe and what's not in terms of physiological breech.* (S113, midwife, colleague)  *I think it's the things that I said bit the team being really visible training between teams and departments kind of being able to ask the tricky questions, circulating research, all of those things that increase comfort and confidence.* (S113, midwife, colleague)  *I feel like we've got quite a strong team, because we've got [OptiBreech staff] and [Mirren] who have a lot of experience.* (S114, obstetrician, specialist)  *I think having the team members as resources is what's more useful because you're more able to do those ad hoc, things are a lot easier to do to just you end up having a discussion with someone, and that's like a bit more beneficial and easier to do. And people are more likely to do that thing if just have a conversation with you and then it turns into you going through some things and it's a little bit of like you know on the spot and discussion and teaching where they wouldn't seek out training in their other time, I suppose.* (S115, midwife, colleague)  *So it is important, especially with physiological breech, because whenever you say physiological breech, everybody looks at you as if you are an alien and they don't understand that it's safer than the lithotomy one, you know, until you start. So our plan with Sophie was to start training sessions, for the for the whole team. Uh, so we have postponed it because of circumstances, but we will start them, we will start them hopefully whenever we can the soon as possible. Erm, now ourselves need to keep up to date as well with the training.* (S116, obstetrician, specialist)  *I think we've already started, I think having erm the I think support in the two midwives with the vaginal breech birth that we facilitated really changed the the feel. It completely changed in the way that we've been spoken to about it. Everyone was a little bit more excited than that it can actually happen and that it is a safe option for women. So it was an instant change and I know that we're trying to keep that momentum going* (S116 midwife, specialist)  *We've had most interest from the registrars, really. But it's we we've done the training, but because they’re moving on, they don't feel that they can join trainees. Yeah. Yeah, because they're training. So we've had a lot of a lot of interest from them surprisingly.* (S116, midwife, specialist)  *Yeah, the that positive experience from especially that she's an HCA, in our hospital, erm that experience, but the experience mostly by the midwife who was attending and the band 7. They immediately spread the word that it was it was a great experience and they were they had worries in the beginning but when when it all ended they understood that they worries were false worries, you know.* (S116 obstetrician, specialist)  *Yeah, I think erm just when Dave does his training with the doctors, I do go along and alongside and just discussions I found that I'm receiving a lot more respect from some registrars and maybe some consultants because they could see that I've got the knowledge there and I felt that again with the physiological breech birth and that the midwives, they felt very confident because they knew that I was just there.* (S116 midwife, specialist)  *I think as a midwife when you're trying to discuss the you know these matters with doctors myself, I find that it's kind of brushed off a little bit, it's you know, it's, ohh it's not evidence-based and you've I have to fight a lot harder I think to really get the doctor's engaged and really wanting to you know, work alongside us. So I think that's a little bit different where I think if it's acknowledged, as being a specialist role and know that’s their title, I think that does make a little bit of a difference unfortunately, which, ideally it shouldn't but.* (S116, midwife, specialist)  *I have the hands on clinical experience, you know, not as much as [OptiBreech staff] has or [OptiBreech staff] has taught, you know, because we deliver twins and second twins, you know, because I've had overseas experience, I have that. And I'm a consultant, you know, I can say, I'm confident and people will believe—.* (S118 obstetrician, specialist) |
| **3.0 Clinical Care Delivery** | |
| **3.1 Attending Vaginal Breech Births** | *I've attended nearly 30 vaginal breech births and many more labours.* (S100, midwife, specialist)  *So I'm currently the only person who meets the proficiency criteria. So I've spent a lot of time on call. And that isn't sustainable in the long run. But I'm kind of viewing it as a process. I'm the thin edge of the wedge, if you well.* (S100, midwife, specialist)  *… currently I haven't got a consultant that would be willing to put themselves on call and come in. So that's me doing that.* (S101, midwife, specialist)  *I think at the moment there's not enough, no one else has any real clinical experience of vaginal breech birth. So they've done the training, but because there was so little -- I mean, prior to me joining, as part of the research we went back and there had been two unplanned vaginal breech births in two years, so very, very low numbers. So we've got quite a long way to go, but there are people interested, but at the moment, I'm doing the majority of it.* (S101, midwife, specialist)  *And predominantly, I'm the one with the most experience, so I'm on call with someone else from the team to come and support, and that's kind of the model that we've been going with at the moment.* (S101, midwife, specialist)  *… it's involved a lot of on-call, a lot of on-call time to come in and support the wider team to facilitate births.* (S103, midwife, specialist)  *All women that had a breech presentation were referred in to see me, and I was able to counsel them in all their options, and I would do on calls to facilitate women having a breech birth.* (S103, midwife, specialist)  *But then the rest of the week, completely uncovered. To kind of, try and do my best, I've got my personal number and my work number on labour ward so that they can call me if they get a lady come in with an undiagnosed breech who, you know, wants to try for a vaginal breech. Then they can contact me. And I would come if I'm available.* (S105, midwife, specialist)  *I've done a lot of extra on calls for the women that I've cared for.* (S105, midwife, specialist)  *I think as an obstetrician I have assisted quite a few breech births and I’ve had to do manoeuvres beforehand.* (S108, obstetrician, specialist)  *I think the more breech births we attend in the hospital when we were called in by the obstetrician, and we should be called in by the obstetrician, the more likely we are to be able to be that expert person with that helicopter view really.* (S109, midwife, colleague)  *And I imagine it would be around having a skilled practitioner and not going ahead with a vaginal breech slash physiological breech without a specialist member of the team. And that could be a resident obstetrician or our consultant midwife or others that are linked to her via the study. She's normally on call for planned breech.* (S113, midwife, colleague)  *I mean, so I mean, what I did was I reacted to that, by telling reassuring my consultant colleagues that, you know, you're not going to have to come in in the middle of the night to do loads of vaginal breeches, because I will come in the middle of night to do the vaginal breech. And I don't think they'll be loads. Because if there are loads that will find a solution to this. In time, we'll get a team set up, we'll do whatever. But at the moment, I don't anticipate there's going to be loads. And I will take the burden in this OptiBreech one phase when it's just here, and just having a look and see what the response is like a test of concept kind of bit of the trial. So they were kind of reassured by that bit.* (S118, obstetrician, specialist) |
| **3.2 Antenatal Counselling** | *And then the other thing, what's important to women and why I think they make different decisions when I counsel them, is because I'm saying there's at least a 90% chance I'll be there.* (S100, midwife, specialist)  *She's well informed, it's been discussed. I make sure that when women are planning a vaginal breech birth that we have that counselling as currently with a midwife and an obstetrician and the woman, because I want to make sure that it's collaborative. And therefore, when I'm presenting that to other obstetricians and midwives, that it's not this crazy midwifery thing that these wild midwives go off and do. And actually, everyone was in agreement with this. So it's predominantly led by me, but I get an obstetrician involved just to make sure that it's a collaborative care plan, really.* (S101 midwife, specialist)  *I've got quite an extensive counselling form for women that are having a vaginal breech birth, and I make sure that that's all signed and uploaded onto all of the systems and everything so that people feel safe.* (S101, midwife, specialist)  *I feel quite passionately about supporting women's choices and being really balanced about risk and chance and those kind of things. I'm not a big fan of sort of, high or low risk, I'm really passionate about individualising care.* (S101, midwife, specialist)  *So it's about us building the women's confidence and reassuring them that we've got some expertise and I think that will increase the rates of our vaginal breeches.* (S102, midwife, specialist)  *I feel relatively confident in my knowledge to able to provide unbiased information around choice and around providing any information in a context that women can understand to make that choice. I think because of the job that I do, I'm conscious of the language that I use when I counsel women anyway.* (S102 midwife, specialist)  *I mean, it's not really an ethical service because not everyone is getting the same counselling. So not everybody is being provided with all the options. They're not being provided with evidence-based information. And so it needs to change to ensure that all women with a breech presentation are provided with the correct information around their options for breech birth.* (S103, midwife, specialist)  *So I'm generally interested in ensuring that women have options and real choice in the way that they give birth. And I think that the way that we counsel women about breech in this country and the big push for all women with breech babies to have caesarean sections is robbing women of that ability to have real choice in the way they give birth. And I think that it's about refocusing breech birth as a different kind of normal, not as a outside of normal way to give birth.* (S104, midwife, specialist)  *I think, so the lady that we did have, who had a vaginal breech birth. I met her as a consultant midwife because she had already had one vaginal birth. She didn't see why she needed a section this time. And she said it was, what made all the difference was knowing that there were people on her side.* (S104, midwife, specialist)  *So I think proper, you know, informed choices is the most important thing. And I'm really happy with the women that have managed to get in contact with me and for me to go and counsel them because I've had women who have opted for relaxative Caesarean and about women who have opted for a vaginal breech birth. And I'm completely happy with either choice they made because I know that that they've made an informed decision, and that's the most important thing, really to me.* (S105, midwife, specialist)  *When the woman comes over to the maternity they can, they can see much more confident team and much more supportive team, in their decision, this is the most important. So that the patient's decision is much more supported than it was before.* (S106, midwife, colleague)  *I think she at the moment is doing all of the counselling alongside an obstetrician.* (S108, obstetrician, specialist)  *I would like to see women properly counselled in a way that they are offered evidence in a neutral and scientific evidence based way without being in unconsciously probably pushed towards away or not to a way of giving birth or another. So I like to see more, more proper counselling, like with real evidence based data without putting your opinion into the into the counselling room as a clinician.* (S110, midwife, colleague)  *I think it will provide non kind of judgmental facts and figures to women for them to be able to make an informed decision without being kind of scared into making a decision that they maybe wouldn't have made if they'd have had like a, I don't know what word I'm looking for but, but like, just somebody that is not kind of prejudiced towards vaginal breech birth and can actually counsel them properly.* (S112, midwife, colleague)  *Yeah, yeah, I think it increases the range of choices. And I mean, I think often because people have read stuff on the internet, they come in expecting that caesarean is going to be the only option and then are surprised by the choice of physiological breech. I'm not sure what's going on in general clinics. But normally, I think that's been one of the roles of consult midwife to kind of scoop people up and to give them the full range of information.* (S113, midwife, colleague)  *And then around like full counselling on expectations of physiological breech as well and vaginal breech and making sure that the family is really clear. On what yeah, the, the increased likelihood of resuscitation being needed, etc. So, yeah, it's mainly around really thorough, informed decision making.* (S113, midwife, colleague)  *Yeah, that it's full and that she gives time and they felt like they're really consulted and informed and can make a genuine decision for themselves. So very supported.* (S113, midwife, colleague)  *I've changed the way I counsel people like I mentioned, so I’m more confident discussing physiological breech as an option.* (S114 obstetrician, specialist)  *I think it improves it, in that when we're able to have a conversation with them, we're able to provide, I suppose more balanced counselling in terms of what is likely to occur if you do choose to have vaginal breech birth.* (S115, midwife, colleague)  *So even our experience and counselling has changed. We at the beginning when we had the first couple we were still you know as if we have some kind of dyslexia or something with that but yeah. But but then after that it just became so fluent no. And then we talked to women as if it's something that we do every day and and women see that and they see that we have knowledge in what we're saying and we have confidence and they gain confidence because of this.* (S116 obstetrician, specialist)  *So we have told our colleagues that we have a breech birth trial going on and we would like them to refer the cases to us to counsel. Now a lot of our colleagues are referring women with breech babies. Sorry. And when they when they are happy to have that counselling, we usually do them in our own time, especially [BSM name redacted].* (S116, midwife, specialist)  *And we answer all the questions, so it makes it easier when we when we can provide just counselling for breech*. (S116, midwife, specialist)  *I think it's just that familiarity with the member of staff and just feeling more comfortable and knowing, knowing what’s already been discussed and maybe they've got further questions from previous discussions and that seems to be much better to be found as we've had positive feedback regarding that.* (S116, midwife, specialist)  *When they've had counselling, when they first knew they had a breech baby and then when they come to talk to us, they feel a huge difference with the attitude towards breech. And the way we we give them confidence that they will be supported with their choice. They understand all the risks that we talk about, but maybe mainly because we have more time for that, we provide more that more time. And antenatal clinic, especially if it's a crowded clinic, you can't give more than like 10 to 15 minutes if that if that's possible to you know to talk about the whole condition of the woman's pregnancy, let alone just about breech. But when they come, we usually give them enough time and well, all the time they need.* (S116, midwife, specialist)  *So I think they could see how much the counselling, you know, how much we invested into the counselling and how confident the woman was, you know, in her body and how much she enjoyed the experience that really positively impacted the unit.* (S116, midwife, specialist) |
| **3.3 Presentation Scans** | *And there's some sort of historical thing going on at [name redacted] which I still haven't got to the bottom of, where a number of senior midwives were considered proficient to do basic presentation scans. But then that was overturned and the ability to do that was taken away. And then I think someone re-instituted that but didn't realise there were all these historical people who used to have it, as a competency.* (S100,midwife, specialist)  *So there's all these people who can scan but aren't technically allowed and I'm trying to navigate that because the provision of a service isn't just about your intrapartum breech skills. So if they want to be part of the whole care pathway, they need to be able to scan. And so that's my next thing on the agenda, trying to get them signed off so that they can provide some antenatal care.* (S100,midwife, specialist)  *At the moment, it's an obstetrics led service, just the ECV part, do you know what I mean? And that is because I haven't done my sonography training, and the Trust is reluctant for midwives to scan without having completed some formal training.* (S101,midwife, specialist)  *I still haven't got actually done the course, the scan course, so part of the stuff what needs to be done like the scan, so the midwife from day assessment was helping me.* (S106, midwife, specialist) |
| **3.4 ECV and Other Methods of Baby Turning** | *At the moment, it's an obstetrics led service, just the ECV part, do you know what I mean? And that is because I haven't done my sonography training, and the Trust is reluctant for midwives to scan without having completed some formal training.* (S101, midwife, specialist)  *So our consultants -- that's why we've got about four consultants who are going to be on board, and they will sort of do one in four of the ECV clinics if women want an ECV.* (S102, midwife, specialist)  *….and then we'll run like an ECV OptiBreech Clinic out of our birth centre. So our consultants -- that's why we've got about four consultants who are going to be on board, and they will sort of do one in four of the ECV clinics if women want an ECV.* (S102, midwife, specialist)  *Thinking about consultants, it's about -- at the moment the ECVs are just brought into Labour Ward and whoever's on the Labour Ward does the ECV, for example, and then they get re-counselled if it doesn't work, if it does work.* (S102, midwife, specialist)  *I think the midwife should be able to do the ECVs, I think it should be a midwife-led ECV service as well. And obstetricians can would also do ECVs, but maybe for those that haven't been successful first time, the second attempt can be by an obstetrician.* (S103, midwife, specialist)  *Women have longer to get -- come to terms with having a breech baby and also try alternative methods for turning their baby, we know that they work better earlier on.* (S103, midwife, specialist)  *So, yeah, the hope is going forward, we'll have a midwife-led breech clinic. With a doctor working alongside who would train the midwives to do ECVs.* (S105, midwife, specialist)  *So when a woman has a breech suspected at 34 weeks, then the community midwife will give her an information leaflet. And that information leaflet has information about postural techniques to help turn baby into a cephalic presentation. It gives information about our acupuncture service, which we can do acupuncture and we can provide the moxa sticks for moxibustion for women to try at home as well.* (S105, midwife, specialist)  *So if there, if there's a lady who midwife or one of the obstetric doctor team find out that there's a breech presentation, so she is referred straight the breech clinic, and she's obviously seen by [OptiBreech staff]. So [OptiBreech staff] she is responsible for the counselling, and the rest of that procedure she's doing like ECV.* (S106, midwife, colleague)  *I think, it is much more put in the effort, it is, I need to just, apart from the cost of what I have to do that, the scan, the moxibustion and be, you know, and learn much more about the breech, even though I have done that course, I still need to come back and, and learn and remind myself, it's not helpful that we don't have so frequently the patients who can just help our experience to develop much quicker.* (S106, midwife, colleague)  *I’d love there to be a member of the breech team for ECVS and being able to be more.* (S108, obstetrician, specialist)  *I think a lot of women would probably be counselled against a vaginal breech, certainly by the registrars on the day assessment unit, not -- potentially by the consultants who do the ECVs.* (S108, obstetrician, specialist)  *We do have midwives in the trusts that are trained in things like moxibustion, but they're not able to administer moxibustion. They're just allowed to advise the woman and explain how it works. And the woman can then choose to do that. And other alternative therapies that we can, if midwives are trained, they can't give it but they can recommend or discuss alternative therapies such as acupuncture. And there's also a lot of discussion, particularly the homebirth team, of spinning babies as well, the spinning babies website. So position changes in pregnancy.* (S109, midwife, colleague)  *I've already mentioned I see a lot of women because I do ECV.* (S114 obstetrician, specialist)  *And I also talked to them about spinning babies. So we do a lot of asking people to do spinning babies before ECV as well.* (S114, obstetrician, specialist)  *Jess is a Midwife… she has a clinic of women that she sees, and then equally if we have ladies that come in for ECV… so our ECVs are performed on the labour ward at [S117].* (S117, midwife, colleague) |
| **4.0 Education and Training** | |
| **4.1 Organising Formal Training Sessions** | *It's my responsibility to lead the team and develop skill within the team.* (S100, midwife, specialist)  *But again, like they've begun to take up some of those teaching opportunities like teaching someone about the video, that's included in doing repetitive reviews and teaching other people. So they're getting really close.* (S100 midwife, specialist)  *I do the vast majority of the teaching for breech skills at [S101]* (S101, midwife, specialist)  *And then we've got about four or five other midwives that did the training. Actually, we've got another obstetrician that comes to do the teaching sometimes.* (S101, midwife, specialist)  *And some more of the team are going to start coming to shadow teaching with me so that they keep those skills up as well.* (S101 midwife, specialist)  *… making sure that the right people have been given the proficiency questionnaire, that the right people have got access to the training, which the Moodle would help that, and it's been challenging identifying consultants that are willing to be to participate. Because obviously I am expecting them to do sort of six or seven and a half hours of CPD. I'm also expecting the team to do six to seven and a half hours of CPD, which I'm not going to be able to give them the time for so this relies on them doing it in their own time*. (S101, midwife, specialist)  *So I teach on the maternity education days.* (S101, midwife, specialist)  *So went straight back from that, rejigged all of our mandatory education for our multi-professional team and introduced physiological breech birth for the whole multi-professional team.* (S103, midwife, specialist)  *… its mandatory training for all our obstetricians and midwives so everybody has to attend every year. They have to have an update on vaginal breech birth, so the same opportunities are there.* (S107, midwife, colleague)  *…the implementation of training for all members of staff as part of mandatory training.* (S109, midwife, colleague)  *And then following on from that then developed kind of more more midwives in the hospital, and the implementation of training for all members of staff as part of mandatory training. Then we introduced the OptiBreech online Moodle package. And then that led to the WhatsApp group and the OptiBreech team meetings. So that was kind of a natural progression to that point. And now I think the team are in a really good position where we've had some, we've got some breech births under our belt. So we've got some experiences that we can talk about. And we can relate to.* (S109, midwife, colleague)  *So much better, the OptiBreech training, because the mandatory training is a station amongst that all other emergencies that we do in one day. So everything is concentrated in one day. So we have to go through very important emergencies in a little like very little time usually is an hour. The OptiBreech training was, I don't remember, but it was, I believe, was half day.* (S110, midwife, colleague)  *So, to work it into prompt training, but not so much as an emergency, but as a variation of normal, which still needs to be handled with care and attention.* (S113, midwife, colleague)  *So like I said, we've had it on our prompt study days. So that's definitely changed training.* (S114, obstetrician, colleague)  *So my role is that I’ve attended some of the training that is led by a midwife called [Jess]. She has done some training with us all, alongside our erm, we do prompt emergency training and emergency drills training. She offered the training alongside that.* (S117, midwife, colleague)  *I’ve also undertaken, I don’t know, it might even be OptiBreech led, I’m sure it is as she’s very keen on it, erm, like an online training package, like lots of videos and then questions following each video. Erm, I’ve undertaken that. The only issue we’ve had with that is just recently changed over IT systems, or probably maybe 12 months ago, erm, at work, so a lot of the, across the board, it’s not just OptiBreech but all videos with mandatory training, you can get the training but the videos won’t play. Erm, they’ve been working on it, but it’s just not quite got there yet.* (S117, midwife, colleague)  *….moving forward, it will be having that consistently and permanently, almost that the OptiBreech team can always have access to those resources because it could be six months between having one vaginal breech and having another.* (S102, midwife, specialist).  *What I need to do is I need to do all my delegate lists, and I need to do all of the people involved in the trial, I need to put all those together. And then the ones that have done the training I was going to send them the competencies of ones that had completed the training.* (S102, midwife, specialist)  *I think from the from the actual midwifery OptiBreech team's perspective, so our home birth team, what they find really beneficial are like the videos and those little things that we've been doing like the episiotomy one, and that those things are -- they've been really positively received. Some of them who haven't been able to access it would love to see that recordings of those because, especially when you know, you've got a breech birth coming up, it's coming up to 40 weeks or at term they are using a lot of the resources to almost revisit and, revise almost, I suppose -- because even though they do the training, it doesn't mean that you can do it -- we haven't still haven't done lots and lots and lots of vaginal breech births, but they feel having access to that at the drop of a hat, access to the videos, especially they find really useful to watch that mechanism, to then think about the shoulder press, think about the buttock lift all of that type of thing, they find really, really useful to revisit.* (S102, midwife, specialist)  *I'm envisaging it's going to take a number of months before the team really feel that they are able to support other members of the team, as it did myself, although interestingly for me, it didn't come from facilitating the birth so much, it came from doing the teaching. And I have regular opportunities to provide training because I was teaching with [OptiBreech staff] at the time, and that's really what I think gave me my confidence and skill because I was continually practicing those new skills and teaching them, that they -- when it came to facilitating a birth, I didn't even have to think about what it was I was going to do, it just, I knew because I had been -- it became second nature almost. And that's, that's what takes time. So I think it's going to be providing those teaching opportunities for the teams so that they can really have those, -- know those manoeuvres and know those signs that something is not right and they need to act on and make it second nature for them.* (S103, midwife, specialist)  *The team, I mean, I definitely -- moving forward with the team, I am asking people to complete the online training. I've sent out the survey. We've had a few people complete that survey, but I sent that out probably a week ago, and it needs to be followed up.* (S103, midwife, specialist)  *And for the people that haven't had that, so the -- most of the newer midwives, Agata, who's the PDM, is doing hands-on skill sessions, which once we have a woman who wants to have a vaginal breech birth we'll offer those more regularly. So in the past, we've when we've had a planned breech birth waiting in the wings, we've kind of tried to put on two sessions a week so people can drop in and and refresh themselves on what to do. Yeah, so that's kind of the model we're going for at the moment*. (S104, midwife, specialist)  *We have most of us who have attempted, have had done some part of the training, if not all, some people have done all of the training, some people have done 50%, 75%, 25%. And, and so it's good now it's something that we can actually relate to, you know, it's not just kind of this concept, you know, we can, it's relatable, we can talk about the experiences we’ve had, what we can learn from the experiences that we had, how we can improve our practice. So yes, I think the OptiBreech team is coming along nicely. But I think there has to be that continual trickle of information giving and sharing of experiences to keep that kind of keep that going. (*S109, midwife, colleague)  *So we've only just started the team this literally this week, we got the email saying this, you guys have been chosen for the OptiBreech team. And this is kind of what we would expect of you. And so so far, we've kind of set, [OptiBreech staff] kind of organized all of us to have like a refresher training, which we've got, I've got mine tomorrow.* (S112, midwife, colleague)  *And that's where the team is helpful because you're hopefully all saying the same thing because we're all trained in the same way and we're all coming from the same viewpoint. So, you know, I mentioned about jobs for emergency section. So if we can get, make sure that a woman who comes in with a breech in labour, whether it's known about in advance or undiagnosed, can see one of the breech team so that we're consistently saying the right thing and you've not got that kind of throw of the dice of who you happen to see on that day.* (S114, obstetrician, specialist)  *So alongside my consultant midwife colleague who is also in the team, the pair of us worked together to do the training in the team when we have the team meetings.* (S115, midwife, colleague)  *Erm, I feel like theoretically I feel very confident, I feel like you know, I’ve read it a lot, I've taught it a lot, and the teaching of it, is the thing that really that sort of helps embed the theory as opposed to just learning it yourself. Teaching other people is what really solidifies it for me in terms of being confident in the theory of and being able to explain and demonstrate to people.* (S115 midwife, colleague)  *And so what we've also done is we've just had our new intake of doctors. So we put on the physiological breech training afternoon for them to kind of go through it. So we're just going to try and bring it in that way, of doing kind of regular skills and things, and addressing consultant concerns separately because I think they probably wouldn't want to speak out quite so much in front of junior doctors. So, yeah, we're kind of taking it from providing lots of training and information for them.* (S104, midwife, specialist)  *I also took over the training for the trust for maternity staff for breech.* (S105, midwife, specialist)  *When we were ready to launch the team, we did have [OptiBreech staff] come up to do a site visit and provide a day's training. So that was an opportunity that came about, which was really useful for the midwives in the breech team and some other midwives that attended as well. And it's kind of allowed us to get a, the OptiBreech training package that we've now got so that's more comprehensive than what we were teaching before.* (S105, midwife, specialist)  *It's it's a bit shame that we haven't done any training sessions for midwives, but I'm sure that after the first training session we will have a lot of enthusiasts. Because at the moment they say, oh, I'm interested, I'm interested. Send me the link, we'll go on online training and we'll all of that. And then nothing that happens, we do send them the links, but nobody logs on. And but if we go ahead and do workshop with--, in the room, training, classroom training and so on. I’m sure that we will have a better response.* (S116, midwife, specialist)  *So I I do run, um, breech training for trainees every like 2-3 months.* (S116, midwife, specialist)  *So it is important, especially with physiological breech, because whenever you say physiological breech, everybody looks at you as if you are an alien and they don't understand that it's safer than the lithotomy one, you know, until you start. So our plan with Sophie was to start training sessions, for the for the whole team. Uh, so we have postponed it because of circumstances, but we will start them, we will start them hopefully whenever we can the soon as possible. Erm, now ourselves need to keep up to date as well with the training.* (S116, obstetrician, specialist)  *We just want to, to see how the training goes because we are planning on putting like, I don't know, six sessions a year probably for breech training to to get everybody involved as many as we need* (S116, midwife, specialist)  *…so she’s, she teaches very well, which I think is helpful. I mean everybody will have sat through training where you just sort of feel yourself glazing over, but she’s not, she’s quite, erm, interested in that she has lots of real world experience which always speak well to, midwives I think really respond well to that, you don’t want really want to learn from somebody who is just reading out to you, you could do that yourself.* (S117 midwife, colleague) |
| **4.2 Conducting Birth Reviews with the Clinical Team** | *I've also done in one of our breech meetings I did a kind of reflection on that birth just so the other members of the team could kind of learn from that birth as well.* (S105, midwife, specialist)  *So I think I've kind of mentioned the support, having one person having oversight over all women, and having regular communication between members of the team and regular meetings for reflection. I think those the main things I think are really important to keep that service going.* (S109 midwife, colleague)  *… we can talk about the experiences we’ve had, what we can learn from the experiences that we had, how we can improve our practice. So yes, I think the OptiBreech team is coming along nicely.* (S109, midwife, colleague)  *I think the immediate thing was, how can we learn? So you know, we all came together, and the midwife spoke about her experience. And it was all about what can we do to learn and move forward? So it was very, it was very, although it was really awful and difficult, it was a very good kind of progression really in, in the team, you know, although there was that feeling of oh, gosh, that that didn't go so well. And there was already kind of right, what can we learn from this straightaway? So so that was really good and positive that we were able to get together as a team.* (S109 midwife, colleague)  *And I also have like [OptiBreech staff]'s contact details and things like that. And she's always said to me that she's very happy for me to contact her and discuss any case, any situation I can call her whenever if somebody comes in, and I think something's been done wrong. And she can call in kind of support me through it.* (S112 midwife, colleague)  *Then, because I've got a lot more confident, I had a lady come in when I was on call over Christmas, and I felt confident enough to do a physiological breech birth with her. Which there was, like one point, I needed to do something and I did feel a bit like, “Oh, my goodness, did I do the right things,” but I did a little kind of reflection and debrief with [OptiBreech staff].* (S114, obstetrician, specialist)  *And I said to [OptiBreech staff], afterwards, oh, my God, you know, what should I have done? I can't believe it was the perineum, holding it back all this time that that was just ridiculous. And she said, “Well, it delivered straight after you did the episiotomy, so you obviously did the right thing.”* (S114 obstetrician, specialist)  *The other thing that I found quite helpful is, I suppose debriefing or discussing the births that we have had with each other, so the the learning can be shared with the rest of the team, so making sure I mean the one birth I said I went to was not a straightforward breech birth, it was a little bit complicated and to be able to for me as this person who attended the birth to be able to go and speak to one of the OptiBreech team members and go through what I did and what happened and for them to sort of reflect with me about, you know, what potentially could been done differently or whatever else was really beneficial for me in terms of my learning, but then for us to think, “OK, well, how can we actually change anything off the back of that,” erm, it was useful to take it to a team meeting and say, “Oh, you know well, I've discussed this on a 1 to 1 basis,” which is more appropriate for me in terms of what I needed following that birth and then for the team we can then discuss it now that we've had that conversation and say, actually, you know, here's a case study to go through and share and learn from, and I think that was really helpful for the team.* (S115, midwife, colleague) |
| **4.3 Facilitating Informal Training** | *And that is one thing that I really liked is that the film was made and some of the people on the breech team even though they weren't at the birth, it's like they're taking ownership because it's one of our births. And so some of the senior midwives have started sharing it with the team at the beginning of the shift and just drip drip dripping. So there is a sense of a learning culture beginning to develop.* (S100, midwife, specialist)  *And again, because there is this sense that doctors teach doctors and midwives teach midwives, I think some of the doctors have been reluctant to come in and learn about the breech births that are occurring. But in terms of just being with this team, like the WhatsApp, we keep sharing information there. So we're sharing all of the things that we're learning constantly and even people who are not directly involved in care are getting experience about the cases that are going on and I think that is helping, yeah.* (S100, midwife, specialist)  *… inviting anybody to access the education because we've had quite a few people that are interested that wouldn't necessarily want to be the on-call side of things, but actually still want to develop their breech birth skills and undertake the education, which I think is great. So if it's on our Moodle platform, then it means that they can do it, because I can't provide them time to do it, because I don't have any more uplift. But they can do it in their own time. And obviously, because of the trial, it's free to access so it's funded effectively, so that would be useful.* (S102, midwife, specialist)  If you've not had the training or the reflections or an experience, then, you know, your confidence is likely to knock off, I think it's just about that constant kind of offer of communication and training. And yeah, I think that's, that can be one of the barriers really, is, yeah. (S109, midwife, colleague)  *I've told them they need to come to one in three meetings just to keep their skills and their knowledge up to date and, you know, to share learning and things like that. So my role as a leader has kind of developed, I suppose, and I'm learning a lot about leading a team through doing this.* (S105, midwife, specialist)  *I think it's the things that I said bit the team being really visible training between teams and departments kind of being able to ask the tricky questions, circulating research, all of those things that increase comfort and confidence.* (S113, midwife, colleague)  *I think having the team members as resources is what's more useful because you're more able to do those ad hoc, things are a lot easier to do to just you end up having a discussion with someone, and that's like a bit more beneficial and easier to do. And people are more likely to do that thing if just have a conversation with you and then it turns into you going through some things and it's a little bit of like you know on the spot and discussion and teaching where they wouldn't seek out training in their other time, I suppose.* (S115 midwife, colleague)  *Yeah, I think that I think we're, very positively in that I've had, many people approach me to ask about training and, also being an opportunity that, um, once people are aware that you're involved in something to breech, people then like to talk to you about breech and they'll talk about their experiences, and it provides you with an opportunity to do a little bit of ad hoc teaching around, OK, while you tell me about your experience, and I can explain what was happening, you know, physiologically and why you saw what you saw or why what happened happened, or you know why certain things were necessary. And try and sort of help people connect their experience with a little bit of background knowledge in that fashion.* (S115 midwife, colleague)  *And then she’s given us like, she sends every now and then a bit of an email about, “Oh I’ve found this that’s really good to watch” or, things that she, she’s very interested in it, so things that she thinks that the team might take away from it as well.* (S117, midwife, collegue) |
| **4.3 Promoting Practical Exposure to Vaginal Breech Birth** | *And then the other thing, what's important to women and why I think they make different decisions when I counsel them, is because I'm saying there's at least a 90% chance I'll be there.* (S100, midwife, specialist)  *So once we ironed that out and it was made clear that my role was to support her named midwife so that it was safer. I'm the safety net rather than the extra midwife coming in to take care of the breeched baby.* (S100, midwife, specialist)  *And that is one thing that I really liked is that the film was made and some of the people on the breech team even though they weren't at the birth, it's like they're taking ownership because it's one of our births. And so some of the senior midwives have started sharing it with the team at the beginning of the shift and just drip drip dripping. So there is a sense of a learning culture beginning to develop.* (S100 midwife, specialist)  *I mean I think some of them lack confidence, but that would be the people who have attended like four breech births.* (S100 midwife, specialist)  *Like the thing that made me really sad about that birth is that [inaudible] none of the doctors or the senior midwife saw it. So the only people learning from that birth were birth centre midwives who were unlikely to be very involved in breech births again for a long time. And the others didn't come to the birth because it's across the corridor. So we missed out. I mean, as it happens that woman wanted her birth to be filmed so they very much had learned from it. And that is one thing that I really liked is that the film was made and some of the people on the breech team even though they weren't at the birth, it's like they're taking ownership because it's one of our births. And so some of the senior midwives have started sharing it with the team at the beginning of the shift and just drip drip dripping. So there is a sense of a learning culture beginning to develop. And I'm hoping that over time as we have more good outcomes, that more of the doctors will become interested and not see it as something that doesn't have anything to do with them because the midwives are doing it.* (S100, midwife, specialist)  *…yesterday I had an email from a lady that's booked elsewhere that wants to transfer to us because we're running the OptiBreech trial and she feels that she would be given a real choice, and that's not the service that she's been offered at the hospital she's at.* (S101, midwife, specialist)  *So it's about us building the women's confidence and reassuring them that we've got some expertise and I think that will increase the rates of our vaginal breeches.* (S102, midwife, specialist)  *I think from the from the actual midwifery OptiBreech team's perspective, so our home birth team, what they find really beneficial are like the videos and those little things that we've been doing like the episiotomy one, and that those things are -- they've been really positively received. Some of them who haven't been able to access it would love to see that recordings of those because, especially when you know, you've got a breech birth coming up, it's coming up to 40 weeks or at term they are using a lot of the resources to almost revisit and, revise almost, I suppose -- because even though they do the training, it doesn't mean that you can do it -- we haven't still haven't done lots and lots and lots of vaginal breech births, but they feel having access to that at the drop of a hat, access to the videos, especially they find really useful to watch that mechanism, to then think about the shoulder press, think about the buttock lift all of that type of thing, they find really, really useful to revisit.* (S102, midwife, specialist)  *And I would try and make sure that they were caring for the women that came in. It wasn't always possible, but so that the wider team could then gain their experience as well.* (S103, midwife, specialist)  *… it's involved a lot of on-call, a lot of on-call time to come in and support the wider team to facilitate births.* (S103, midwife, specialist)  *So my role was really then gaining my own experience, but also supporting others to gain their experience as well. And that ranged from junior midwives through to senior midwives, through to obstetricians and consultants.* (103, midwife, specialist)  *And moving to [site], I'm now doing a similar thing, attending breech births as the on call person to facilitate the births and support the wider team.* (103, midwife, specialist)  *We're gonna try and sort of buddy up people that have never been at a breech, but need the exposure, with someone that's quite experienced with it. Or, you know, obviously, if [OptiBreech Staff] can come in and offer support on any of the days. Then we sort of try and ensure that if there's someone that hasn't got hands on breech or, you know, maybe it's quite junior in their career that there's somebody a little bit more experienced that's buddying with them, to support them. So that yeah, again, they're getting the exposure but in the sort of safest way, of having somebody with them.* (S104, midwife, specialist)  *And by practising we I think we just can we can create a proper, proper breech team on labour ward.* (S105, midwife, specialist)  *And this is what I think [OptiBreech staff] really helped me to boost in my confidence and I think it's just, this is the part, I know that we, you know, the the patient on labour ward, I don't need the four people, four obstetrics team, I don't need the obstetrics team, I don't need, I know how to conduct a delivery. I know what I'm to do. I know step by step. And this is and gives me absolutely, I could even say that happiness that that I know what to do in this in this case and, and this is the, this is what I actually I can say about the confidence.* (S106, midwife, colleague)  *… with anything you always have a little bit of inside anxiety but you never show that because it doesn’t help with teamworking.* (S108 obstetrician, specialist)  *One of the things that our trust is doing at the moment is, if anyone attends triage, with an undiagnosed breech birth or or planned vaginal breech birth, an obstetrician and midwife and the Optibreech team are going together to the birth so that we can improve and increase our competence and confidence.* (S109, midwife, colleague)  *So I think that you never feel competent until you have experienced something at least once or, or over and over again. So you can do all of the training, you can feel confident in that you know, what you think you're going to do and you know what, you know, the skills and the techniques that you'll need to be equipped for the birth. But I think that until each of us has had an experience with vaginal breech birth that hopefully you know goes well. I think you will never feel truly competent. Like any skill. So I think the more women we recruit, the more women we support, the more likely we are to feel competent and confident.* (S109 midwife, colleague)  *And I think it's needed to have the extra training for staff and, and then being able to see more breeches, and then everybody else feeling more confident, because they see more breeches. And so it kind of has a knock-on effect.* (S112, midwife, colleague)  *And then she's been on call. So I've seen the women come in, and they've called her in and she's been there to support. She hasn't, she hasn't kind of, she doesn't really do the deliveries, herself, she kind of sits there and supports the midwife as well, which I found has really, really helped. So if that continues, with the way it looks like it's going to, with the breech team, that we can support other midwives to become more competent, and doctors to come become more confident and competent. And in doing it, then I think it was going to really, really help.* (S112, midwife, colleague)  *… for example, I had a lady recently that had come in and was breech and started to labour and had had that discussion. And she decided to have a vaginal breech birth. And then the doctors were kind of getting her ready to go into lithotomy. And I came in and I was like, “Well, how about we just try this.” And the doctors were in there. And they were watching and she delivered really beautifully in an upright position. And they all said afterwards that it was amazing. So I think the more they see it, the more comfortable they're going to be with doing it. And so hopefully, then the rates of the upright breech birth will go up.* (S112, midwife, colleague)  *She hasn't, she hasn't kind of, she doesn't really do the deliveries, herself, she kind of sits there and supports the midwife as well, which I found has really, really helped. So if that continues, with the way it looks like it's going to, with the breech team, that we can support other midwives to become more competent, and doctors to come become more confident and competent. And in doing it, then I think it was going to really, really help.* (S112, midwife, colleague)  *Yeah, I do. I think it makes a huge difference if your own consultant midwife is a breech specialist.* (S113, midwife, colleague)  *I think, again, having the consultant midwife there or available? Or indeed, like, just the presence of her in the in the, the team in the senior team does make a difference to people's confidence.* (S113, midwife, colleague)  *I think its about outcomes, I think, the more we can do, and the more we can demonstrate that the outcomes are good, the better.Attending births to support other staff members.* (S114, obstetrician, specialist)  *I think its about outcomes, I think, the more we can do, and the more we can demonstrate that the outcomes are good, the better. Attending births to support other staff members.* (S114, obstetrician, specialist)  *And I had done a couple of physiological breech birth, either by staying with a midwife, who was one of our senior midwives, who was doing some, or I just had a preterm one that I did on my own. It was quite funny actually, because we got to the point where I felt like I needed the buttock lift. And just as I was about to do it, this little voice behind me went I think you need a buttock lift, and there she was, and I didn't even know she was in the room.* (S114, obstetrician, specialist)  *And it's difficult because when a woman does come in for a breech birth, we're already trying to get the trainees and midwives that haven't seen it or experienced it to have some involvement or see and learn from it. So I would say, medical students and midwifery students probably don't get enough of a look in because always there'll be a whole massive crowd.* (S114, obstetrician, specialist)  *And I think it's also very individual basis and it very much depends on if you've had a role model who has spoken positively about breech or if you've not, or you've had some kind of experience of someone who is positive about it compared to if you don't.* (S115 midwife, colleague)  *The difficulty with the breech team is, I suppose, the lack of experience. Which is like the chicken and egg situation of a lot of them, we've done the training, we've done some simulations and everything else but a lot of them haven't seen a lot of real life breech births, so a lot of them they've done the training and not fully sort of competent, because they haven't been to enough breech births and we don't get a lot of people coming through having vaginal breech births.* (S115, midwife, colleague)  *I think for me I'm getting the OptiBreech birth encourages a bit more conversation about breech options and tries to encourage the options to be available and also, trying to provide staff with more confidence to attend a vaginal breech birth intrapartum, because a lot of staff who are attending birth it will be the first time or they haven't seen it before. So being someone who can be there to reassure and gain people positive experience and make it safer because if you have one of us there who knows what to look out for and how to manage things best in a more timely fashion than hopefully you can have better outcomes and better overall experiences for the midwives and for the women and for everyone involved in the birth experience. Really, because a lot of times you hear about it not going that way*. (S115, midwife, colleague)  *…it’s difficult to get the team to enough births to see them to feel more confident to be on call on their own.* (S115, midwife, colleague)  *So one of our consultants who's on the team is our clinical director. Erm, and we both attended a breech birth together, and it was really nice to know there was somebody else there in the room who was OptiBreech as well as just me, so it wasn't all on me because the other birth I went to, it was just me and it was the first time I've been on call for the OptiBreech, so it was doing it for the first time where you're in a position of attending a different role, I suppose, and being the first attendant at birth like you usually are as a midwife, and that was a difficult birth the first one.* (S115, midwife, colleague)  *What we started to do with that on call rota as well as put a first and a second on call. So if there's someone who is available, but they're not necessarily confident to attend the birth yet, but they'd like to be called to attend to observe, to get experience, then we're trying to do it that we sort of buddy people up so that there's more than one person on call.* (S115, midwife, colleague)  *I found that I'm receiving a lot more respect from some registrars and maybe some consultants because they could see that I've got the knowledge there and I felt that again with the physiological breech birth and that the midwives, they felt very confident because they knew that I was just there. They're ready if if anything needed to be helped and you know explaining everything that was happening.* (S116, midwife, specialist)  *And I think if women are aware and empowered, they can then ask for things and but you know, regardless, and we're in the trial information out there, it's the word is out in the community, there's lots of community posting, and word of mouth from what I hear. So OptiBreech has resulted in women being aware of it being an option. And I think as well, midwives and doctors being aware of it being an option. And hopefully, as cases build up and people get more exposed to it, term, vaginal breech birth as a viable option they can then the reputation for vaginal breech birth will also, you know, improve.* (S118, obstetrician, specialist)  *And so obviously, like anything else, it's, it's beneficial to have, a specialised team to look after women to gain that expertise. And to consolidate that expertise.* (S118, obstetrician, specialist)  *…we’re a bit different here because we've got [OptiBreech staff], people come from all over the place and sometimes they get here, like that woman who had the two feet visible, she driven for like 3 hours across London because she wanted to come here. So I think we're quite lucky in that respect. We get quite a lot of women wanting to come here* (S119, midwife, colleague)  *We’ve had lots of support from [OptiBreech staff], who's who worked here and is it on the team, and she's been present at so many breech births now. And yes, she’s had to resolve some complications. Everybody does. But I think that's given her confidence and she just wants to do it and she's really positive, really proactive. And she's a really good role model for everybody else. So, you know, she will take in her student midwife and stuff, and I think that's just amazing because we really need to to make sure that the student midwives are confident going forward because it's, it's up to them really to make a change I think.* (S119 midwife, colleague) |
| **4.5 Personal Skill Development and Networking** | *And that's one of the things I like about my job is that I am constantly learning from women as well.* (S100, midwife, specialist)  *Obviously, we have too many WhatsApps, but you know, “What do people think about this?”, “Have they had that?” or whatever. It might be just nice for us to connect, because I think it can be quite -- there's these little beacons across the country, but there will always be people that are -- not resistant, but concerned about vaginal breech birth. And I think it's important that we support each other really, to say, Actually, what we're doing is the right thing.* (S101, midwife, specialist)  *I would still do an on call element. Actually, I would like to still do that because I obviously need to maintain my skill as well.* (S103, midwife, specialist)  *So my role was really then gaining my own experience.* (S103, midwife, specialist)  *But as it had done each birth, my confidence has grown massively. Because you learn with every birth that you do.* (S105, midwife, specialist)  *…there’s considerable hours on my part and maybe the principal investigators part to undertake the e-learning package.* (S107, midwife, colleague)  *And I know [OptiBreech staff]'s been sending out invites to webinars. And it's really sad because I haven't been able to make any single one. But not that I don't want to be that I absolutely do. I would really like to be discussing and questioning and thinking with teams from other units. It's just so far, the times haven't worked for me for various reasons. So I think just that general support, supportive environment in which to discuss and learn together, I guess, to increase confidence of us all.* (S114 obstetrician, specialist)  *And then I did my training because OptiBreech team was being set up. I then did the full online video training thing as part of the setting up and I want to be part of the team. I think I did that in around November, time, or maybe early December. Anyway, I felt a lot more confident after doing the training. And so since then, I've been much more confident in my counselling.* (S114, obstetrician, specialist)  *…. ourselves need to keep up to date as well with the training.* (S116, obstetrician, specialist)  *And in in terms of the network, you know, just chatting to other people involved in the trial via the webinars, or she's got a kind of WhatsApp group with other midwives in it, you know, that I can tap into for just network expertise whenever I'm not sure about something because that's kind of what you want. I think. As a clinician, you know, when I don't feel that I can ask my local network my my usual networks, consultants, because they're not interested or experienced in vaginal breech birth.* (S118, obstetrician, specialist) |
| **5.0 Research** | |
| **5.1 Recruitment** | *But the clinic does make research a lot easier because not everyone who is known to be breech passes through.* (S100, midwife, specialist)  *…yesterday I had an email from a lady that's booked elsewhere that wants to transfer to us because we're running the OptiBreech trial and she feels that she would be given a real choice, and that's not the service that she's been offered at the hospital she's at. I've had several feedback from women that ultimately have decided to have a Caesarean or ended up with a Caesarean that felt so delighted that they just had the opportunity to talk through their choices and felt that they were involved and included in their care. So that's been really, really good. And that's really satisfying to me as a midwife.* (S101, midwife, specialist)  *Look, we're part of this big research, and actually, other women from other Trusts want to come to our Trust because we're offering that.” So I think that all of these small things are good for me to then be able to share and say, “Look, we're doing a good thing here.* (S101, midwife, specialist)  *So then I get the referral, and then I tried to call the women because I know that immediately they're going to start to go home and Google everything, and I've got a list of resources that I send them.* (S101, midwife, specialist)  *So if I've had a lady like most recent lady that wants to be part of it and I said, I think I've got my first recruit. And it's like the questions I asked you before we started this meeting. What's that link? I need the link so that I can consent her because I thought I'd have to do that in person, because, when you do your DCP stuff. So it's those types of things. So it's great to have that you guys to support that definitely.* (S102, midwife, specialist)  *I think the difficulty with recruiting women at the moment is that because we've got a new pathway, nobody is following it at the moment.* (S105, midwife, specialist)  *So we have told our colleagues that we have a breech birth trial going on and we would like them to refer the cases to us to counsel. Now a lot of our colleagues are referring women with breech babies. Sorry. And when they when they are happy to have that counselling, we usually do them in our own time, especially [BSM name redacted].* (S116, midwife, specialist) |
| **5.2 Research Administration** | *So far I have struggled to get people to complete the interest and proficiency surveys.* (S100, midwife, specialist)  *Yeah, not so much. So, actually, I've had very senior support, so the obstetric lead and the head administrative team are supportive of the clinic and supportive of being part of the research. And so that has helped get the clinic off the ground.* (S101, midwife, specialist)  *But it's a balance, in my team in general, we don't have any admin support, and something like that would really help me keep on top of that stuff. It's not my strength, to be honest, data collection.* (S101, midwife, specialist)  *So, for example, getting the data, making sure that the right people have been given the proficiency questionnaire, that the right people have got access to the training.* (S102, midwife, specialist)  *It's the first time I've been a PI. So that's been a little bit scary and a little bit -- and I have to say I do feel a little bit like a fish out of water. So things like the paperwork side of it, all the logs you've got to keep and making sure that that's all -- all those things are done and that apprehension, I suppose sometimes puts me off a little bit, doing some of the stuff I want to -- to put the service in and I can put a service in. But then doing all the paperwork for the research side of things, like a different entity, making sure that's all done properly. So that's not been easy to navigate. But I suppose it just comes with experience, it's been great to have you and [OptiBreech staff] as contacts.* (S102, midwife, specialist)  *I am asking people to complete the online training. I've sent out the survey. We've had a few people complete that survey, but I sent that out probably a week ago, and it needs to be followed up.* (S103, midwife, specialist)  *So I'm hoping [Agata], who's taking over from me as PI will be supported by the new consultant midwives coming in to make sure those kind of things are done.* (S104, midwife, specialist)  *So yeah, so my focus is around counselling women who want to give*  *birth outside of guidance, and in writing new guidelines, updating*  *guidelines and getting involved in research to bring to the trust.* (S104,  midwife, specialist)  *…this was collective led by our consultant midwife who is the principal investigator.* (S107, midwife, colleague)  *Uh, you know when Nancy was here, everything was really slick and smooth and everything. Now she went then another one took over as interim, and she didn't get the job, she was trying to help as long as she was working, but the help is nothing like what Nancy was doing. Now there's a new one who took over, she never contacted us, I don't know her name yet, and she only started to work last week, probably. So we haven't made contact with her yet.* (S116, midwife, specialist) |
| **5.3 Data Collection** | *I'm trying to capture quite a lot of data and spreadsheets and stuff. I mean, not so much from the trial, because actually, I've done a few of the CRS now, and they are so simple. It took me no time at all, and I could just get all up on clips, I didn't even need to get their forms, and I was like, Oh, that's fantastic. But obviously, whenever I started the new service I was trying to keep track of everyone that was seen in a clinic and what were their outcomes.* (S101, midwife, specialist)  *But it's a balance, in my team in general, we don't have any admin support, and something like that would really help me keep on top of that stuff. It's not my strength, to be honest, data collection.* (S101, midwife, specialist)  *And I also need to then pull off all the data, which I'm waiting for my informatics to get back to me the information you want about two years prior to the trial, and during the trial.* (S102, midwife, specialist)  *So, for example, getting the data.* (S102, midwife, specialist) |
